# Recruitment of a long-term memory supporting neural network during repeated maintenance of a multi-item abstract visual image in working memory

**DOI:** 10.1038/s41598-021-04384-4

**Published:** 2022-01-12

**Authors:** Klaartje T. H. Heinen, J. Leon Kenemans, Stefan van der Stigchel

**Affiliations:** Experimental Psychology, Utrecht University, Heidelberglaan 1, 3584 CS Utrecht, The Netherlands

**Keywords:** Neuroscience, Psychology

## Abstract

Humans can flexibly transfer information between different memory systems. Information in visual working memory (VWM) can for instance be stored in long-term memory (LTM). Conversely, information can be retrieved from LTM and temporarily held in WM when needed. It has previously been suggested that a neural transition from parietal- to midfrontal activity during repeated visual search reflects transfer of information from WM to LTM. Whether this neural transition indeed reflects consolidation and is also observed when memorizing a rich visual scene (rather than responding to a single target), is not known. To investigate this, we employed an EEG paradigm, in which abstract six-item colour-arrays were repeatedly memorized and explicitly visualized, or merely attended to. Importantly, we tested the functional significance of a potential neural shift for longer-term consolidation in a subsequent recognition task. Our results show a gradually enhanced- and sustained modulation of the midfrontal P170 component and a decline in parietal CDA, during repeated WM maintenance. Improved recollection/visualization of memoranda upon WM-cueing, was associated with contralateral parietal- and right temporal activity. Importantly, only colour-arrays previously held in WM, induced a greater midfrontal P170-response, together with left temporal- and late centro-parietal activity, upon re-exposure. These findings provide evidence for recruitment of an LTM-supporting neural network which facilitates visual WM maintenance.

## Introduction

When an artist paints a landscape from life, at first the chosen scene will need to be inspected regularly, but gradually a detailed picture may develop in the painter’s mind. How is the image represented in the brain and how does this representation change, when the artist becomes more familiar with the depicted landscape? The current EEG study investigated whether a shift in mnemonic representation can be observed during learning of an abstract visual scene. Informed by an earlier study, which reported a transition in memory-sustaining neural representations when subjects became gradually more efficient in spotting a visual search target^1–3^, we specifically looked at parietal- and midfrontal components.

The ability to hold representations of several visual items online for direct access and manipulation by higher cognitive functions, is known as visual working memory (WM). There is a capacity limit to the number of items that can be contained in visual WM, which usually sits around 4, but differs across individuals^4^. While traditionally visual WM is thought to rely on a frontal-parietal network, recent evidence suggests that it can rely on MTL/hippocampal structures as well, perhaps particularly when the number of memorized items exceeds the visual WM capacity limit and/or when spatial location is an important dimension of the memory trace (see^5^ for a review). A proven EEG method to measure several aspects of visual WM, such as capacity and filtering efficiency, is the contralateral delay activity (CDA). This is a sustained negativity around parietal-occipital electrodes which scales with the number of memorized items^4,6^ (and see^7^ for a review) and can also be observed while a search template is held online during visual search^8^.

In the previously mentioned study^1^, it was shown that the CDA gradually decreases in amplitude during repeated and increasingly efficient search for the same target in different search contexts^2^. The decrease in CDA amplitude concurred with a negative modulation of the midfrontal P170 component, which was interpreted as a transfer of information between a WM- to a long-term memory (LTM) store. Whether this shift in neural correlates is really linked to an enhanced consolidation of the visual memory trace can however not be concluded from this study, as recognition was not tested afterwards. In addition, a more efficient search performance is likely to reflect (perhaps implicit) short-term strengthening of stimulus–response connections (see^9^), but not necessarily an enhanced *explicit* memory representation.

Negative modulation of the midfrontal P170 component has indeed been associated with long-term (implicit) memory processes such as perceptual priming^10^_,_ involving perceptual categorization and grouping processes^11,12^. Many other studies have implicated a later midfrontal component in conscious recognition^13–16^. In these studies, a dissociation emerges between recognition based on familiarity (knowing), signaled by the midfrontal FN400 component, versus rich detailed recollection, reflected in a later parietal activity^16–25^.

The scalp location of the midfrontal signal (around the Fz electrode) may link this activity with midfrontal theta power (4–8 Hz), originating in the medial prefrontal cortex (mPFC). MPFC theta power modulations have been associated with encoding and retrieval processes in LTM and maintenance during WM^16,26^, possibly through functional connectivity with medial temporal lobe (MTL) and hippocampal structures (see fi.^27,28^ for reviews).

In our paradigm, we aimed to investigate whether a parietal-to-midfrontal shift in neural correlates can be observed when the repeatedly memorized stimulus is not a single search target, but a multi-item colour-array, which needs to be visualized/recollected *explicitly* during the WM task. In our design, the array consisted of more coloured items than can generally be held in working memory, of which one was probed by spatial location-cue during each trial. The array was therefore more akin to an abstract visual scene, which had to be remembered as a whole, rather than a collection of separate targets. However, we expected that initially, participants would encode the coloured items as separate targets in visual working memory, with a gradual transition to a coherent representation of the whole array^29^, which would rely more on LTM sustaining structures.

As in the visual search study^1^, we used the CDA component to trace the working memory representation, while modulation of the midfrontal P170 component was taken as a potential neural signature of memory consolidation within an LTM-supporting network. Bilateral arrays of 6 coloured squares each, were repeatedly presented during a testing block of 8 trials, with one hemifield being cued for covert attention at the beginning of the testing block. In each trial, the colour of one of the squares in the array was probed following a 2.5 s delay, with a combined central colour- and a peripheral spatial-cue highlighting one of the array-squares (‘memory cue’). This way, participants had to construct an explicit mental representation of the colour-array’s spatial lay-out during each trial in order to perform the task. Importantly, the item-set-size exceeded the visual WM capacity limit, which we expected to require stronger recruitment of LTM (MTL/hippocampal) structures (see^5,30^).

During the main experiment, parietal contralateral activity- and midfrontal-responses were measured during the early learning- (trial 1–4) and late learning phase (trial 5–8) of the WM blocks, but also analyzed in a temporally finer grained analysis across four groups of 2 trials. These ERP components were measured from array onset throughout the delay, relative to the ERP in the control attention (ATT) condition, during which the cue was presented simultaneously with the colour-array. The same components were also measured during *recollection/visualization* of the memorized array, probed by the memory cue in the WM condition following the delay. With respect to the parietal component, we refer to contralateral activity (CA) while the array or memory-cue is present on screen and contralateral *delay* activity (CDA) during the WM delay following array-offset.

In a subsequent post-hoc old/novel recognition task memory *consolidation* was assessed. The midfrontal P170 and FN400 components to old- and novel colour-arrays were measured, to assess lasting neural modulation accompanying longer-term implicit- and explicit visual memory consolidation^10,13,14,16^. Importantly, the responses to ‘old’ colour arrays were split into two categories, depending whether arrays had previously been presented during the WM- or the ATT-condition.

During the main experiment, we expected a gradual decline of a WM-specific CDA component, when learning progressed, compared to the ATT-condition. Conversely, we expected an increasingly negative modulation of the midfrontal P170 component during repeated WM^2,3^. We further predicted a more prominent parietal component with gradually more successful recollection/visualization of the colour-array upon presentation of the memory-cue in the WM condition, akin to what has previously been described for explicit LTM recollection^15–25^. Furthermore, we hypothesized that, if the midfrontal response modulation signaled recruitment of a longer-term memory network during the main experiment, this modulation should be predictive of accuracy in the post-hoc recognition task. Consequently, longer-term visual memory is expected to be visible as a lasting neural modulation of the midfrontal P170 response^13–16,31^, especially upon re-exposure to old colour-arrays that had previously been held in WM. In addition to these specific predictions, we also employed a more explorative whole brain analysis during these conditions, inspecting midfrontal- as well as parieto-temporal regions.

## Materials and methods

### Participants

Twenty right-handed healthy volunteers (sample size based on an expected change in the CDA difference wave of ~ 1.5 µV; with desired power of 90%) with normal (or correct-to-normal) vison and no reported colour-blindness upon inquiry, were recruited to take part in the experiment (11 female; age 25.5 ± 6) and were financially reimbursed for their time. All participants provided informed consent and all experimental methods conformed to the ethical principles of the Declaration of Helsinki and were approved by the Ethics Committee of the Faculty of Social and Behavioural Sciences of Utrecht University.

For the main experiment, all participants were included in the behavioural analysis and one participant had to be excluded from EEG analysis, due to excessive artefacts (see criteria below). For the follow-up recognition task, one participant had to be excluded from the EEG analysis, because she could not complete the task and the ERP analysis was performed on 19 participants. Due to a technical error, behavioural data were not properly recorded for the first 6 participants. The behavioural analysis for this part of the experiment was therefore performed on 14 participants.

### Behavioural task

**Figure 1:**
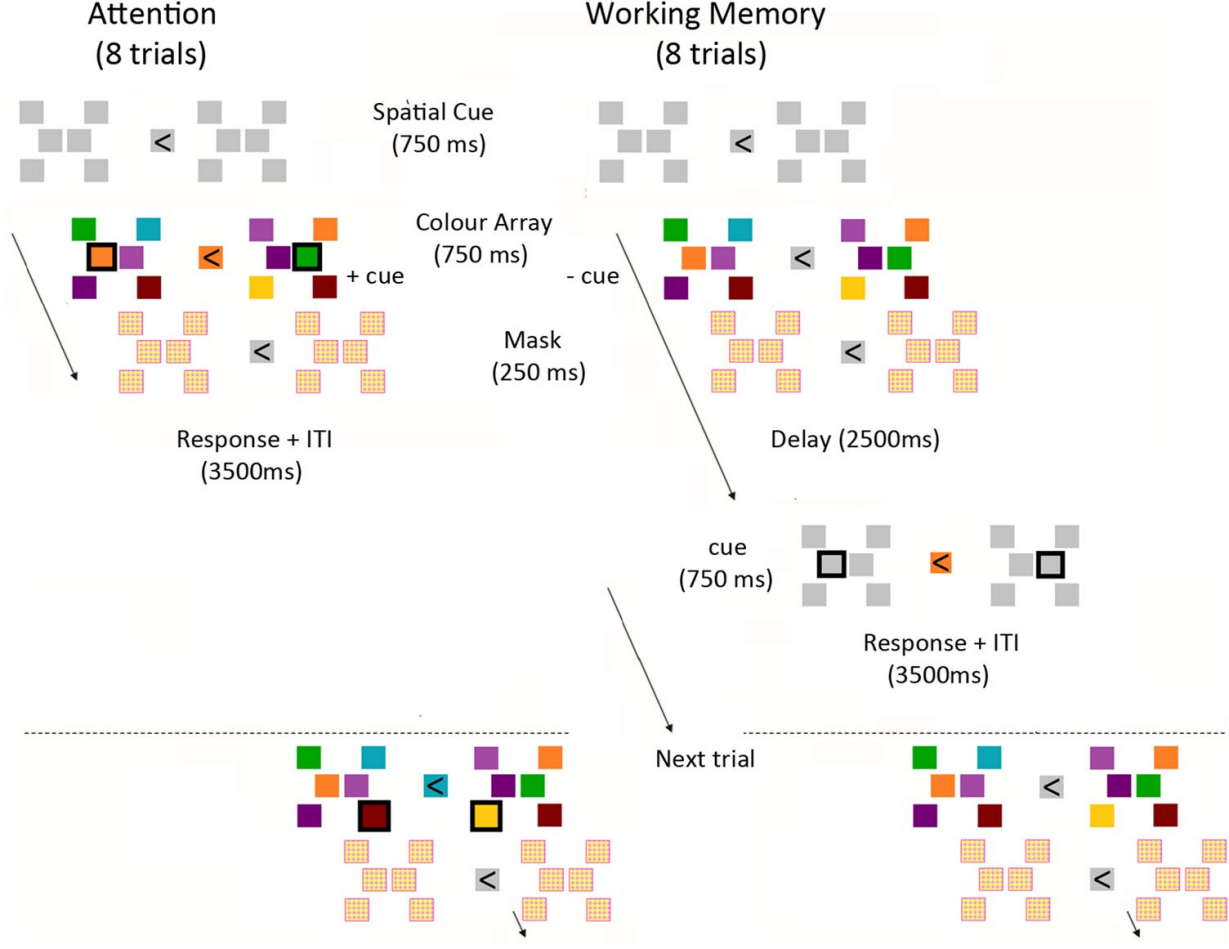
Schematic representation of the task. Two different arrays, consisting of six coloured spatially-fixed squares each, were bilaterally presented with one hemifield cued for covert attention at the beginning of (and throughout) a testing block. The same array-combination was repeated for 8 trials in one testing block and spatial locations of all squares would remain visible as white placeholders during stimulus intervals. Participants had to match a central colour cue with that at one highlighted square in the array, either while the colour-array was still present on screen (attention/ATT condition, left) or following a 2.5 s delay (WM condition, right).

The experiment consisted of two parts, the main experiment and a subsequent recognition task. A schematic representation of the experimental paradigm during the main experiment is shown in Fig. 1. The task (Working Memory (WM)/Attention (ATT)) was manipulated within participants across 8-trial blocks. At the beginning of each testing block, a central arrow indicated which hemifield to attend to during the subsequent 8 trials and would remain visible throughout the duration of the testing block. After 750 ms following spatial cue onset, two different arrays, each consisting of six coloured squares at fixed locations, were bilaterally presented (750 ms), followed by a scrambled colour mask (250 ms). The mask was added to avoid the continuation of neural activity induced by the colour-array immediately after disappearance from the screen (iconic memory). This was an initial presentation of the colour-array-combination that was subsequently repeated in the ensuing 8 trials. A trial would start with a slight brightening of the central fixation square (750 ms). During each trial, participants had to match a central colour probe with the colour at one square in the array, highlighted by a black rectangle around it. This combined cue (central colour and peripheral rectangle) was presented for 750 ms, either while the array was still present on screen (ATT block) or following a 2.5 s delay (WM block). Locations of all squares in the bilateral arrays and at central fixation remained visible as white placeholders at all time when the colour-array was not on screen. ITI intervals were 2.5 s for both conditions. Participants were encouraged to maintain a visual memory of the cued colour-array during the delay periods of the WM condition.

Participants responded with their right hand by using self-chosen keys on a keyboard for “match” or “no-match”. The central colour cue had to be the same as the colour *at the highlighted location* in the array to be deemed a match (50% of trials) and was considered a non-match when the colour was not present in the array at all (25% of trials) or present but not at the highlighted location (25% of trials). The task was non-speeded and participants were instructed to respond as accurately as possible.

Task (ATT or WM) and attended hemifield were randomized across testing blocks. Three match-conditions (match (colour *and* location), no-location-match and no-colour-match) were pseudo-randomized across trials, with a 50/25/25% chance distribution for each condition across each trial position (1–8) within a testing block. Each participant performed 6 separate sessions, comprising 6 testing blocks of 8 trials, with 288 trials in total.

Stimuli were generated on a Dell computer (Dell, Round Rock, TX, USA) and presented on a LG 24MB65PM LCD monitor (2560 × 1440 pixel spatial resolution and 100 Hz refresh rate), using Matlab (Mathworks, Natick, MA, USA; version: R2016a), Cogent 2000 (developed by the Cogent 2000 team at the WCHN and the ICN, London) and Cogent Graphics (developed by John Romaya at the LON at the Wellcome Department of Imaging Neuroscience, London). The size of the screen was 50.8 × 33.9 cm.

The 6 different colours allocated to each square of an array were randomly drawn (separately for each hemifield) from a pool of 8 highly discriminable but equi-luminant colours (red, gray, brown, green, purple, turquoise, orange and pink (24.5 ± 2.5 cd/m^2^). Arrays were presented on a gray background (72 cd/m^2^). Squares in the arrays subtended 0.75° in visual angle each with 2 squares presented in 3 rows, with 1° vertical distance between squares (center to center) and 3° horizontal distance between squares in the upper and the lower row and 1° horizontal distance between squares in the middle row. Centre of the arrays were presented 4.5° from fixation.

Preceding onset of the EEG experiment, participants completed 2 practice blocks of each task condition. During the EEG experiment, lights were switched off, while participants were seated in a comfortable chair, 66 cm from the monitor with their heads resting on a desk-mounted chinrest. They were instructed to maintain fixation on a central square in the middle of the monitor array, while covertly attending the cued hemifield throughout each testing block. Adhered fixation was monitored with an Eyelink1000 eyetracker (SR Research). Participants were instructed to refrain from blinking as much as possible until the 5 s breaks in between testing-blocks.

Following a break of approximately 5 min after completion of the main experiment, during which participants remained seated and attached to the EEG apparatus, the post-hoc recognition task would commence. Participants were not informed about this task until after they had completed the main experiment. For this task, all colour-arrays that had been presented during the main experiment in the attended hemifield (either during ATT- or WM blocks) were randomly intermixed with novel array configurations made up out of the same colour pool. In total there were 72 trials, of which 36 contained novel- and 36 contained old arrays (18 presented during either condition (ATT/WM)). Note that due to randomization of all trials, there was on average an interval of approximately 35 min between initial array-exposure during the main experiment and the test probe during the post-hoc recognition task. A trial would commence with a slight brightening of the central fixation square (500 ms), followed by a central presentation of a colour-array (2000 ms), with an equiprobable chance to be drawn from the ‘old’- or ‘novel’ set. Participants were asked to indicate whether they recognized the presented colour-array and instructed to respond ‘yes’ when they felt more than 60% confident. Responses had to be indicated with their right hand by pressing either of two self-chosen keys on the keyboard. Participants were instructed to withhold their responses until offset of the colour-array and to be as accurate, not as fast as possible. The following trial started after a 3.5 s inter-trial-interval, during which a white square would remain visible at fixation.

### Behavioural data analysis

#### Main experiment

Behavioural measures, including accuracy scores (d’ scores ^32^ during the EEG experiment) and RTs were each analyzed by a repeated measures ANOVA with two factors: Task (Attention/WM) and Trial Group (either: first 4 trials/second 4 trials or: first, second, third and fourth 2 trials). As it was a non-speeded task, accuracy scores were our main measure of interest.

#### Post-hoc recognition task

Sensitivity scores for old/novel discrimination (d’ score;^32^) were calculated for the post-hoc recognition task. The d’ score was calculated using the following equation: d’ = Z (hit rate) − Z (false alarm rate).

Initially d’ scores were calculated including all ‘old’ array trials, irrespective whether they had been presented during the ATT or WM condition during the main experiment and in a subsequent analysis, d’ scores were calculated separately for old arrays that had been presented during the ATT or WM condition.

### Eyetracking

Saccades were detected offline with the default values of the EyeLink algorithm for saccade detection applied across trial segments restricted to the stimulus duration (either 2000 ms following colour-array onset or 1000 ms following memory-cue onset). Trials with saccades during the selected time segments were excluded from further analysis.

Average fixation was within 1.5° from fixation for all participants in all conditions (see Supplementary Fig. S3). Across participants, a slight hemifield bias was observed during the first 4 trials of the WM condition and not in any other condition (Interaction Task × Trial Group × Hemifield F(1,18) = 7 *p* = 0.02). However, this bias was only minor (< 0.3°; see Supplementary Fig. S3e) and we do not expect that this had any substantial impact on the results.

### EEG recording

The EEG was recorded continuously using a 64-channel Biosemi amplifier system with electrodes positioned according to the 10–20 international system (AEEGS, 1991). Separate electrodes were placed on the supraorbital and infraorbital ridges of the left eye and on the outer canthi of the right- and left eyes to calculate EOG signals for blink- and eye-movement artifact detection.

During EEG recording, electrode impedances were kept below 20 kΩ. Each electrode was measured on-line with respect to the CMS/DRL electrodes producing a monopolar channel. The ongoing brain activity at each electrode site was sampled using the Actiview application at 2048 Hz and filtered with a low-pass filter of 100 Hz, and a high-pass filter of 0.16 Hz.

### EEG processing

EEG data were analyzed in MatLab using SPM and FieldTrip^33,34^ software. The data were re-referenced off-line to the algebraic average across all electrodes and then low-pass filtered (40 Hz) to exclude high-frequency noise and high-pass filtered (0.1 Hz) to eliminate slow drift (1 Hz for the post-hoc recognition experiment).

The continuous EEG was then segmented into epochs, time-locked to the onsets of the colour-array. Epochs were 3700-ms long, beginning 200 ms before array-onset and ending after 3500 ms, just before either the onset of the following trial (ATT condition) or presentation-onset of the memory-probe (WM condition). The segment containing the response to the memory probe was epoched separately, starting 200 ms before cue-onset and ending 1200 ms later, 250 ms post cue-offset. The pre-stimulus baseline of 200 ms was used for all analyses.

Data quality was initially inspected using automated algorithms. Epochs containing excessive noise or drift (± 80 μV) at any electrode were marked and channels in which more than 20% of the epochs were affected were excluded. One participant was excluded, of whom more than 20% of the channels was deemed bad according to these criteria. Two participants showed particular drift in frontal channels during the delay period. In these cases, the high-pass filter was adjusted to 1 Hz for analysis of the frontal channels and a more lenient channel exclusion criterium was used (40%). Blinks were identified as large deflections (± 4 × SD) in the VEOG signal (subtraction of the signal from the EOG electrodes above and below the left eye) and were marked. All marked artefacts were later accounted for during robust averaging. Epochs were averaged according to task (ATT/WM), trial group (either: first 4 trials/second 4 trials or: first, second, third and fourth 2 trials) and attended visual hemifield.

### EEG analysis

#### Main experiment

For the C(D)A analysis, ERPs were averaged across the PO7/O1 electrodes (left hemisphere) and PO8/O2 electrodes (right hemisphere). ERPs were subsequently combined across trials by an averaging procedure that preserves the electrode location relative to the attended hemifield (contra- or ipsilateral)^4,6,35^. Previous studies investigating CDA generally studied relatively short delay intervals (ca 300–1000 ms following stimulus onset, see^1,2,4,6^). We therefore initially limited our analysis for these previously reported time-windows. Since stimulus presentation was longer in our paradigm (compared to previous studies) only the first 300 ms post colour array-*offset* was included (reflecting true delay-activity). Mean amplitudes of the difference waves between contra- and ipsilateral delay activity were computed during this 800–1100 ms time-window.

The mid-frontal component was computed by averaging ERPs across 4 mid-frontal electrodes (aFz, Fz, F1 and F2) and mean amplitudes were calculated during the peak response following array onset (P170, see^2,10^) and also during the 300 ms post array-offset.

During memory-cue presentation, parietal CA was measured from the moment it emerged (350 ms following cue onset^4,6,7^) until 100 ms pre cue-offset. The P170 component was measured during the peak, 170 ms following cue-onset.

For the 2 × 4 trial-group analysis, two-way repeated measures ANOVA were conducted on the mean amplitudes, testing the effects of Task- and Trial-group. For the 4 × 2 trial-group analysis, first a one-way-ANOVA was performed on the between-task differences, including all four trial-groups. Post-hoc pairwise comparisons were subsequently performed to identify significant differences between specific trial-groups. Least significant differences-correction was applied, unless the 1-ANOVA had a *p*-value > 0.1, when Tukey–Kramer correction was used.

Relations between the measured EEG modulations during the WM-delay and task-performance were tested twofold. First, the averaged signal (across 1000 ms post array-offset) was compared between correct- and incorrect trials, by applying a 2-way ANOVA including Accuracy and Trial-group as factors. Second, to test whether individual differences in measured signals predicted WM and/or recognition task performance, Robust Pearson correlations were computed between mean amplitude and task performance (d’). We performed this correlation analysis on the longer time-window into the WM delay (1000 ms following array-offset). For completeness, both analysis were also performed on the short 300 ms time-window.

The percentage bend correlation is a robust method that protects against outliers among the marginal distributions (Matlab Corr_toolbox 2012; Cyril Pernet and Guillaume Rousselet 26-01-2011). Trimming percentage, i.e. negative weighting of outliers, was set to default (20%).

The Greenhouse–Geisser epsilon correction for nonsphericity was applied to all ERP analyses where appropriate^36^, and only corrected probability values and degrees of freedom are reported.

Topographic current–density T-maps displaying differences between conditions were shown to illustrate ERP-effects or as additional explorative analysis (such as the investigation of recollection/visualization-associated activity post memory-cue presentation, see Fig. 5 in the main text). In all cases cluster-based permutation tests were used (Monte Carlo) to obtain statistics from the distribution and cluster-correction was applied^37^.

#### Post-hoc recognition task

The analysis of the post-hoc recognition task aimed to test longer-term visual memory. Potential modulation of the midfrontal P170 response to repeated (old) colour-arrays, separated in arrays that were previously shown during the ATT- or WM-condition, compared to non-repeated (novel) colour-arrays was measured^10^. As longer-term plasticity effects were not necessarily expected to occur exactly during the peak of the response, the P170 signal was averaged across a longer time-window (170–270 ms).

## Results

To test whether a learning-associated shift in visual WM correlates could be observed during repeated encoding and recollection of a multi-item colour-array, we conducted an EEG experiment using a visual WM paradigm as depicted in Fig. 1 (and see “Materials and methods” section). This was followed by an array-recognition task to test consequences of neural modulations for longer-term consolidation.

In a previous pilot experiment we had shown that gradual learning of the colour-array configuration reached a plateau performance after approximately 8 trials (see Supplementary Fig. S1b). Moreover, the array-recognition task in this pilot experiment confirmed longer-term memory consolidation, in particular for colour-arrays presented during the WM- as compared to the ATT condition (see Supplementary Fig. S1c). In a second pilot experiment, we tested the hypothesis that a set size exceeding the visual WM capacity-limit (6) would enhance the reliance on a longer-term memory-supporting storage system for holding a mental representation online during visual WM, which should be reflected in a stronger memory consolidation. We found indeed support for this hypothesis, as 6-item arrays were better remembered in a post-hoc recognition task than 4-item arrays (see Supplementary Fig. S2).

### Behavioural performance during the EEG experiment (WM task)

**Figure 2:**
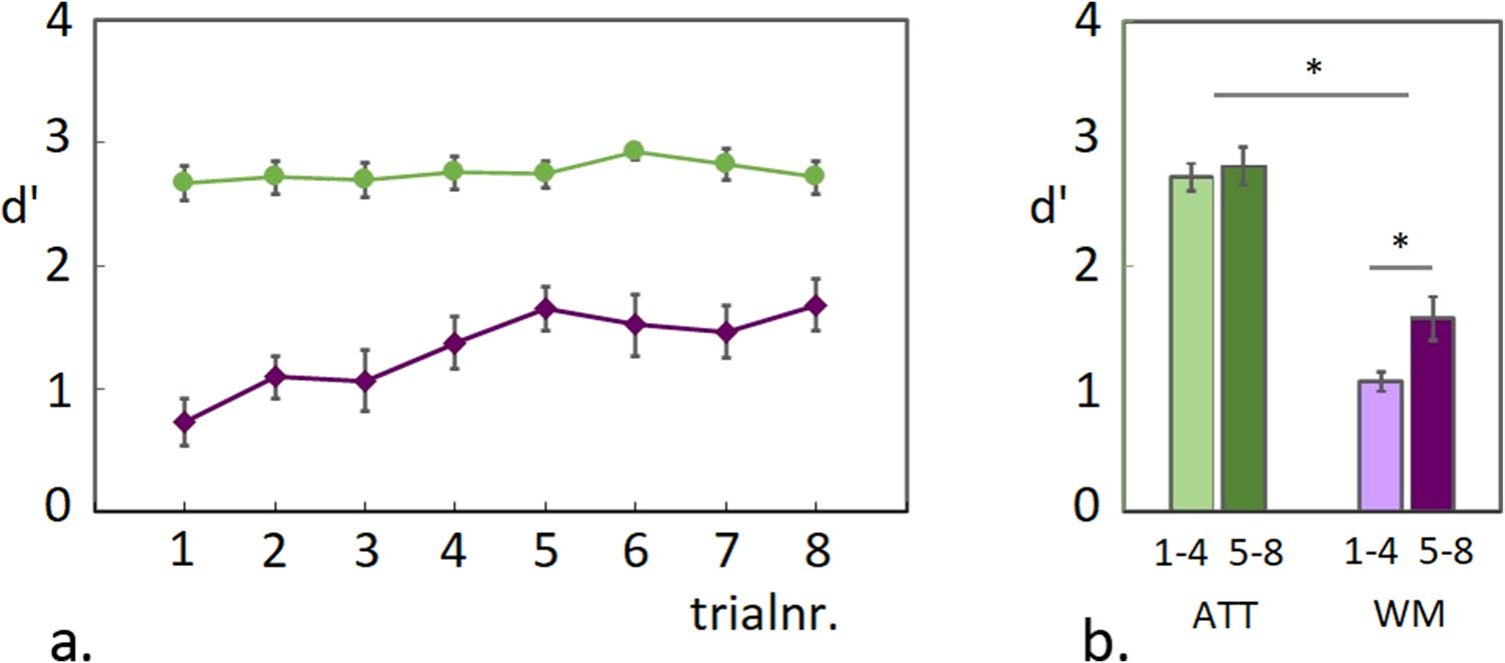
WM task accuracy scores. (**a**) Mean d’ scores during the ATT and WM conditions are plotted for 8 consecutive trials in a testing block. A gradual increase in accuracy during the WM condition reached a plateau around the fifth trial, while performance for the ATT condition was around ceiling throughout the testing block. (**b**) Plotted are the accuracy scores for both conditions, averaged across the first- and the second four trials.

Guided by the pilot findings, 6-item colour-arrays were used in the EEG experiment, which were repeated during 8-trial blocks. A similar gradual increase in accuracy-scores over trials was observed in the EEG experiment, as had been shown for 6-item arrays in the second pilot experiment (see Fig. 2a and Supplementary Fig. S2). For statistical analysis of task performance, accuracy scores (d’) were collapsed across the first- and the second 4 trials separately and an ANOVA was applied with Trial-group (first 4 trials/second 4 trials) and Task (ATT/WM) as factors. As shown in Fig. 2b, the gradual increase in performance during the WM condition was reflected in a significant difference between trial-groups, while no significant change was detected during the ATT condition (Main effect Task F(1,19) = 19 *p* < 0.001; Main effect Trial Group F(1,19) = 25.3 *p* < 0.001; Interaction Task x Trial-group F(1,19) = 15.6 *p* = 0.001; pairwise comparison between trial-groups for the WM condition T(1,19) = -5.1 *p* < 0.001 and for the ATT condition T(1,19) =  − 1.7 *p* = 0.103 ns.). This observed increase in accuracy scores for the WM condition did not coincide with faster reaction times (see Supplementary Fig. S4).

As outlined before, we expected that this gradual learning across trials in the WM condition would be accompanied with a parietal-to-midfrontal shift in neural correlates during the memory delay period^1,2^. Specifically, we predicted that a parietal CDA component would be most prominent during the first 4 trials (early learning), when we expect the coloured squares to be held as separate targets in working memory. This should decline when learning progresses and a more profound modulation of the midfrontal component was expected to emerge during the second 4 trials (late learning), presumably signaling a transfer to an LTM-supporting storage system.

### Behavioural performance during the EEG experiment (recognition task)

The main task was followed by a post-hoc recognition task, during which all colour-arrays that had previously been presented in the attended hemifield during the main task (ATT- or WM condition) were shown again, randomly intermixed with novel array configurations, made up out of the same colour pool. Participants had to indicate whether they recognized the presented arrays with a confidence higher than 60%. Note that due to a technical error, behavioural data for this part of the experiment was collected for 14 participants.

**Figure 3:**
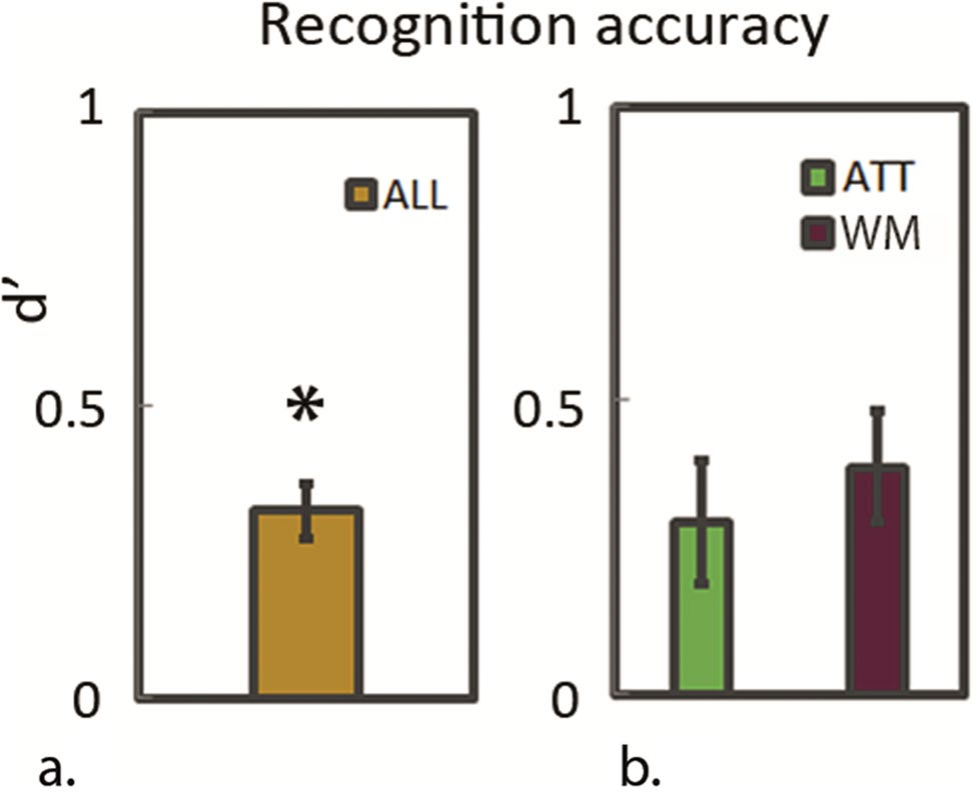
Recognition task accuracy scores. Accuracy scores on the post-hoc recognition task are plotted as d’ scores across conditions (**a**) and separated for colour-arrays that had previously been shown during the ATT or the WM condition (**b**).

As observed in the behavioural pilot experiments preceding the EEG study, the repeated exposure to the colour-arrays resulted in a positive d’ score during the post-hoc recognition task as plotted in Fig. 3a (mean d’-score = 0.32; T(13) = 6.73 *p* < 0.001). In contrast to the first pilot experiment (see Supplementary Fig. S1c), no significant difference was detected between d’ recognition scores for arrays shown during the ATT- versus the WM condition (Fig. 3b; pairwise comparison T(13) =  − 0.53 *p* = 0.6 ns.). It should be noted however, that during the earlier behavioural pilot, experimental conditions were slightly different and had more trials per block (see “Discussion” section). However, also with 8 trials per test-block in the EEG session, we observe a modest but significant above chance d’ score across conditions in the recognition task, indicating longer-term consolidation across conditions.

### EEG analysis: encoding and maintenance during the visual WM task

#### Parietal contralateral activity

**Figure 4:**
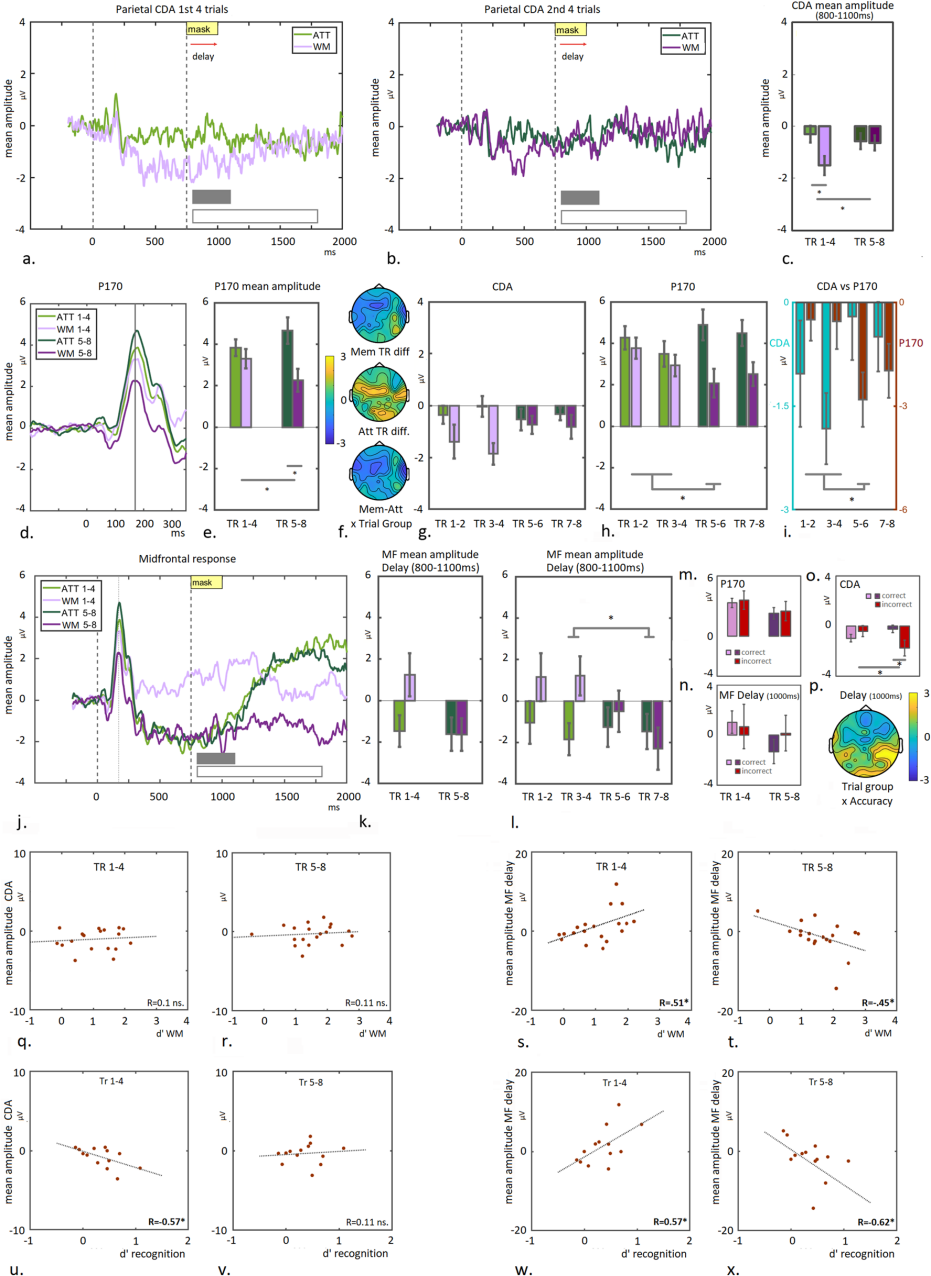
Parietal contralateral activity and midfrontal response during encoding and WM maintenance. Upper panel: C(D)A components are plotted as difference waves between contra- and ipsilateral activity per task-condition (ATT/WM), from the moment of colour-array onset and throughout 2000 ms of the WM delay period for the first 4 trials (**a**) and the second four trials (**b**). The mean amplitudes of the C(D)A difference waves are plotted per task-condition and trial-group, as calculated during a 300 ms time-window immediately following array-offset (**c**; time-window indicated as filled gray box in **a** and **b**). Second panel: Plotted are the midfrontal responses for all four conditions during 350 ms following colour-array onset to show the P170 peak (**d**). Mean amplitudes per task-condition and trial-group are plotted for the P170 peak (**e**; time-point indicated as gray line in **d**). Current density T-maps display the trial-group differences per task-condition and their interaction (**f**). Mean amplitude per task-condition split into four two-trial-groups for CDA (**g**) and P170 (**h**) Task-differences per trial-group are plotted for each component (**i**). Third panel: Plotted are the midfrontal responses for all four task conditions from colour-array-onset throughout the WM-delay period (**j**). Mean amplitudes per task-condition and trial-group are plotted for the 300 ms time-window immediately following array-offset averaged across four trials per trial-group (**k**) and 2 trials per trial-group (**l**). Mean amplitudes are plotted for correct- and incorrect trials in the WM-condition for the P170 component (**m**) as well as CDA (**o**) and the midfrontal component during the WM-delay (**n**), both averaged across 1000 ms post display-offset (indicated by open gray box in **a**, **b** and **j**). Current density T-map of the Trial-group × Accuracy interaction during the WM-delay (**p**). Lower panels: Correlations are shown between mean amplitudes during the WM-delay (CDA and midfrontal responses averaged across 1000 ms post display-offset) and d’ accuracy scores during the WM task (CDA: **q**, **r**; midfrontal: **s**, **t** ) and the post hoc recognition task (CDA: **u**, **v**; midfrontal: **w**, **x**), asterisks indicate *p* < .05.

First, the parietal contralateral response was measured from colour-array onset (Contralateral Activity, CA) and throughout the memory delay or ITI (Contralateral Delay Activity, CDA) per task condition (ATT/WM) and trial-group (first 4 trials/second 4 trials). This response was plotted as difference waves between contra- and ipsilateral activity across occipito-parietal electrodes (see Fig. 4a, b). As can be seen in Fig. 4a, a C(D)A component was visible for the WM condition, starting around 300 ms post array-onset and persisting during a short time into the delay period during early learning (first 4 trials).

As a CDA component was only detectable for a relatively short time during the delay, even during the early learning trials, we calculated mean amplitudes during a selected time-window of 300 ms following array offset (plotted in Fig. 4c; see also “Materials and methods” section) and compared these between both trial groups to test our prediction of a learning-associated decline in the CDA. An ANOVA, including the factors Task and Trial-group, revealed a significant Task x Trial-group interaction (F(1,18) = 4.7 *p* = 0.045; pairwise comparison between-task for the first 4 trials T(18) = 2.76 *p* = 0.013; pairwise comparison between-task for the second 4 trials T(18) = 0.13 *p* = 0.9 ns.; pairwise comparison between-trial-group for WM-condition T(18) =  − 2.13 *p* = 0.048 pairwise comparison between-trial-group for ATT-condition T(18) = 1.09 *p* = 0.29 ns). In other words, while a WM-specific CDA component was present for 300 ms following array-offset during early learning (first 4 trials), it had disappeared during late learning (second 4 trials). It has to be noted here that for the late learning trials, a Contralateral Activity (CA) was still visible during array presentation. Qualitatively, the decrease in strength of the C(D)A component when learning progressed, seems therefore to reflect a narrowing of the response for late learning trials to a short time-window during array presentation*.*

In summary, as predicted, we observe a learning-related decline in the WM-specific parietal C(D)A response following array onset. However, even during the early learning phase (first 4 trials), this component is only visible (averaged across participants) for a relatively brief time into the WM delay period (but see also below). Note that the signal was corrected for saccades and blinks up till 1 s post array-offset, which may account for a noisier signal later during the delay.

#### Midfrontal response

Next, it was tested whether learning of the colour-arrays in the WM condition concurred with a negative modulation of the midfrontal component, which has previously been associated with long-term perceptual priming^1^. The midfrontal component was averaged across four electrodes (AFz, Fz, F2 and F3) per task-condition (averaged across hemifield) and per trial-group, and plotted for the first 350 ms following array-onset to visualize the P170 peak (see Fig. 4d), as well as from array-onset throughout the WM delay (see Fig. 4j). Mean amplitudes were calculated for all four conditions for the P170 peak (see Fig. 4e) and, as for the CDA analysis, also for the 300 ms time-window post array-offset (see Fig. 4k).

An ANOVA, including the factors Task and Trial-group, revealed a main effect of task for the P170 component, with a smaller amplitude during the WM- compared to the ATT condition (F(1,18) = 23.2; *p* < 0.001; see Fig. 4e). The negative modulation of the P170 peak in the WM condition became more pronounced when learning progressed, as compared to the ATT condition. This was reflected in a significant Task × Trial-group interaction, with an enhanced difference between WM and ATT for the second 4 trials compared to the first 4 trials (Task × Trial-group interaction: F(1,18) = 4.65; *p* = 0.045; pairwise comparison between-task for the first 4 trials: T(18) = 0.999; *p* = 0.33 ns.; second 4 trials: T(18) = 4.54; *p* < 0.0001; pairwise comparison between trial-groups for each task condition did not reach full significance: ATT T(18) =  − 1.75 *p* = 0.10; WM T(18) = 1.48 *p* = 0.156). Current density T-maps showing between-trial-group differences for both task conditions and their interaction (lower panel) are presented in Fig. 4f. As can be seen, the minimum of the negative modulation between trial-groups during the WM condition is centered around the Afz electrode (upper panel), with an opposite pattern slightly more posterior electrodes during the ATT condition (middle pannel).

Mean amplitudes during the 300 ms time-window post array-offset are shown in Fig. 4k. A 2 × 2 ANOVA did not reveal a significant Task × Trial-group interaction effect and marginal main effects (Task × Trial group interaction: F(1,18) = 2.3 *p* = 0.147; Main effect Task (F(1,18) = 3.9 *p* = 0.06; Main effect Trial group F(1,18) = 3.5 *p* = 0.078). Note that direct comparison between the task conditions is slightly problematic here, since the ATT condition required a button-press immediately following array-offset (this confound is canceled out in the difference potential of the CDA, but not for the MF analysis). The comparison of interest is the pairwise comparison between trial-groups for the WM condition, which did not reach full significance (T(18) = 1.77 *p* = 0.093). However, when splitting trials into four two-trial groups to get a more fine-grained temporal impression (TR1-2, TR 3-4, TR5-6 and TR7-8; see also below), a one-way ANOVA between trial-groups revealed significant differences between TR1-2 and TR7-8 and between TR3-4 and TR7-8 (resp. *p* = 0.021 and *p* = 0.019; corrected for least-square differences; one-way ANOVA for between task-differences across trial-bins F(3,72) = 2.58; *p* = 0.06 see Fig. 4l).

In summary, we observe an opposite pattern during learning for P170- compared to CDA modulation. While the CDA-component was only present during the first 4- and not during the second 4 trials, the P170- and sustained midfrontal signal modulation was most pronounced during the second 4 trials.

### Comparison of temporal pattern between CDA and P170

To obtain a better insight in the relation between temporal changes in the CDA and P170 components, trials were grouped in four bins of 2 trials for both components (see Fig. 4g and h) and the differences between task-conditions were directly compared between CDA and P170 (see Fig. 4i). A 4 × 2 ANOVA revealed a significant difference across trial-groups (F(3,72) = 3.6 *p* = 0.018). Post-hoc pair-wise comparison specified the significant differences to occur between TR1-2 and TR5-6 (*p* = 0.022), between TR3-4 and TR5-6 (*p* = 0.005) and between TR3-4 and TR7-8 (*p* = 0.04; least significant differences correction for multiple comparison).

A 2 × 2 ANOVA between the two middle trial-groups (TR3-4 and TR5-6) confirmed this Component × Trial-group Interaction (F(1,18) = 8.4 *p* = 0.01). Looking at the Task × Trial-group interaction for the CDA and P170 components separately, a 2 × 2 ANOVA (including Task and Trial-group (TR3-4 and TR5-6) as factors) revealed a significant interaction-effect for the P170 component (F(1,18) = 5.3 *p* = 0.034) and a marginal effect for the CDA component (F(1,18) = 4.0 *p* = 0.061).

Subsequently, one-way ANOVAs were performed between trial-groups per task and for each component separately. This analysis revealed a significant difference between TR1-2 and TR5-6 for the P170 component during the WM-condition (*p* = 0.05; one way ANOVA across time-groups F(3,72) = 2.6 *p* = 0.06). No such differences were found for the ATT condition (F(3,72) = 0.14 *p* = 0.9). Also no significant differences were found for the CDA component (*p* > 0.26). This may be due to an insufficient number of measurements per condition as the CDA analysis required trials to be separated between hemifields (in contrast to the analysis of the midfrontal signal). Inspecting the EEG traces however, a pattern was observed with an emerging CDA during the first trial-group, becoming most prominent during the second trial-group and which was no longer observed during the last two trial-groups (see Supplementary Figure S5).

In short, this temporally finer grained analysis qualitatively confirmed the previously described decrease in CDA component during the WM condition following the first 4 trials, with this component being most prominent during trial-group two (TR 3-4). The P170 component displayed an opposite and more gradual modulation across the first three trial-groups during the WM condition.

### Functional relevance for behaviour

Next, an analysis was performed to investigate the functional relevance of these findings for task-performance during WM. In a first analysis, responses to correct- and incorrect trials are compared (on average 26% of the WM trials were incorrect). In a second analysis, accuracy is correlated with the measured components across participants. These analysis were done by averaging components across a 1 s period during the delay-period, to investigate longer-lasting effects that may not have been visible due to variation across participants.

### Correct- versus incorrect trials

As mentioned, the previous analyses were all performed on correct-trials only and a similar analysis was now also applied to incorrect trials (for the WM task). A 2 × 2 ANOVA (Trial-group × Accuracy) was computed for each component separately. This analysis revealed an interaction-effect for the CDA component (F(1,18) = 5.84 *p* = 0.026) see Fig. 4o; see Supplementary Figure S7 for applying this analysis on the mean amplitudes of the 300 ms window post array-offset). Pairwise comparison revealed a significant difference between correct- and incorrect trials for the second 4 trials (pairwise comparison: T(18) = 2.2 *p* = 0.046). This reflected something interesting: a CDA component was still observed during the second 4 trials, but only for incorrect trials (see Supplementary Figure S7). An opposite pattern was observed during the first 4 trials (pairwise comparison T(18) =  − 1.56 *p* = 0.135; but see also Supplementary Figure S7). This suggests that the CDA component is beneficial during early learning, but detrimental during late learning.

A 2 × 2 ANOVA (Trial-group x Accuracy) for the P170 component did not reveal a significant effect (*p* = 0.9 ns. See Fig. 4m), suggesting that the modulation of the P170 was not influenced by task-accuracy. A similar analysis on the midfrontal component during the delay revealed no significant interaction when averaging across the 1000 ms period (F(1,18) = 1 *p* = 0.33; see Fig. 4n; but see also Supplementary Figure S7 for a remarkable pattern immediately following display offset). A current density T-map showing the Trial-group × Accuracy interaction during the delay-period however, did show a pattern of maintained negative modulation of activity recorded at the midfrontal electrodes and -interestingly- also showed an enhanced activity above right central-parietal and temporal electrodes (see Fig. 4p).

### Correlations with behaviour

To investigate whether inter-subject variability in modulation of the CDA- and midfrontal components during the WM-delay had any predictive value for task-performance, robust correlation analysis were performed, which compared EEG-responses during correct trials with WM-task performance per participant.

CDA amplitudes were averaged across 1000 ms into the WM-delay and correlated with task accuracy (d’) during the WM task. To test whether the CDA response during the WM delay had any relevance for longer-term memory consolidation, we also performed an additional correlation analysis with recognition task accuracy (d’) (Note that task-performance on the post-hoc recognition task was only correctly recorded for 14 participants, of which one was excluded from EEG analysis of the main task; see “Materials and methods” section).

No significant correlation was found between CDA mean amplitude and WM performance (see Fig. 4q–r; first 4 trials: R = 0.10; T(18) = 0.41; *p* = 0.68 ns.; second 4 trials: R = 0.11 T(18) = 0.47 *p* = 0.64 ns.) In contrast, for the first 4 trials, a significant correlation was observed between CDA mean amplitude during the WM-delay and subsequent performance (d’) during the later recognition task (see Fig. 4u; R = − 0.57 T(12) =  − 2.31 *p* = 0.04), which was not observed during the second 4 trials (Fig. 4v; R = 0.11 T(12) = 0.36 *p* = 0.72 ns; Williams’s test between correlations T(12) = 2.18 *p* = 0.05). Note for completeness: A similar pattern was observed for the 300 ms time-window post array-offset (800–1100 ms): no significant CDA-behaviour correlations were observed with WM accuracy (*p* > 0.6 ns.), but a similar significant correlation was observed with recognition accuracy during the first 4 trials (R = -0.65; T(12) = -2.87; *p* = 0.015). No significant activity-behaviour correlations were observed for the ATT condition (*p* > 0.35 ns.).

The same analysis was performed for the midfrontal component to investigate whether inter-subject variability in modulation of this component during the WM-delay, had any predictive value for WM task-performance (and hence any functional relevance). As for the CDA component, the mean amplitude of the midfrontal signal was averaged during the first 1000 ms of the delay for each participant per trial-group and correlated with individual WM performance (see Fig. 4s, t). This analysis revealed a significant negative EEG-behaviour (d’) correlation during the second 4 trials (R =  − 0.45; T(18) =  − 2.1; *p* = 0.05; Fig. 4t) and surprisingly, a positive correlation was observed during the first 4 trials (R =  − 0.51; T(18) = 2.50; *p* = 0.025; Fig. 4s; Steiger’s test between correlations z(18) = 2.98 *p* < 0.05). These findings indeed suggest a functional relevance of the observed sustained midfrontal modulation for visual WM maintenance.

Subsequently, to test whether the modulation of the midfrontal component during the WM delay phase was predictive for longer-term consolidation (and hence indicative of the recruitment of an LTM supporting system), correlations with performance (d’) on the post-hoc recognition-task were performed. Indeed, significant correlations were observed between the midfrontal mean amplitude during the WM-delay and recognition task d’ (see Fig. 4w, x; first 4 trials: R = 0.57; T(12) = 2.32; *p* = 0.04; second 4 trials: R =  − 0.62; T(12) =  − 2.65 *p* = 0.022), with again a difference between early- and late learning trials (Williams test between correlations t(12) =  − 3; *p* < 0.05).

Similar but not always significant correlations were observed for the short 300 ms segment post array-offset (EEG-WM d’ first 4 trials: R = 0.24; T(18) = 1.0; *p* = 0.31; second 4 trials: R =  − 0.58; T(18) =  − 2.95 *p* = 0.01; EEG-LTM d’: first 4 trials: R = 0.55; T(12) = 2.2; *p* = 0.05; second 4 trials: R =  − 0.53; T(12) =  − 2.08; *p* = 0.06).

No significant EEG-behaviour correlations were observed for the P170 peak (*p* > 0.25 ns).

### EEG analysis: Recollection/visualization during the WM task

#### EEG analysis: visualization during the WM task

Next, we assessed learning-associated changes in the parietal CA-response and the midfrontal P170 component during explicit recollection/visualization of the learned colour-array, as probed by the memory-cue in the WM condition following the 2500 ms delay.

**Figure 5:**
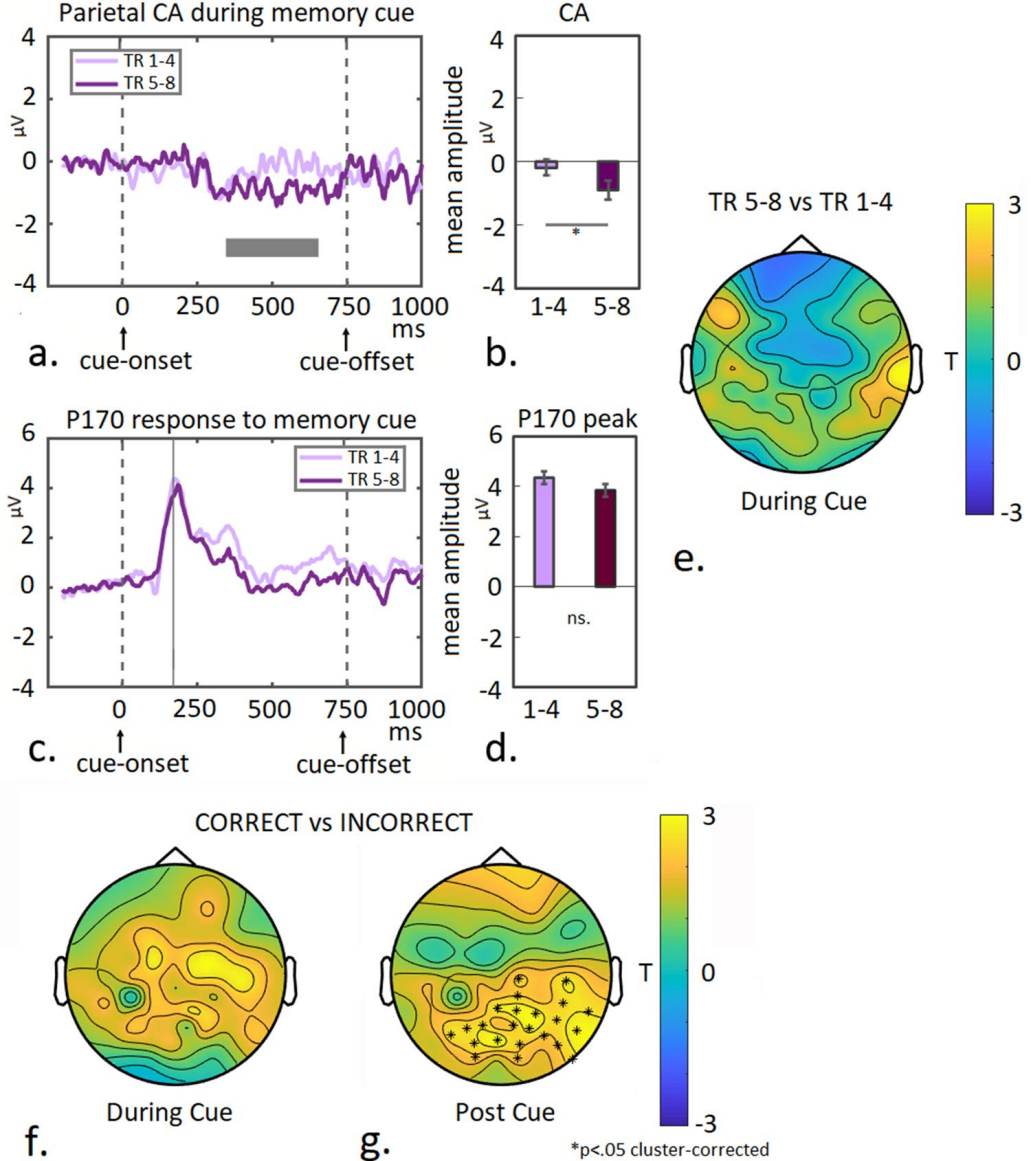
Parietal contralateral activity and midfrontal response during WM-cue-induced visualization. Plotted are the parietal CA difference waves (**a**) and midfrontal P170 responses (**c**) for both trial groups during recollection/visualization of the memorized colour-array, as probed by the memory-cue following the WM delay (t0 = memory-cue onset). Mean CA amplitudes are calculated and plotted for a 300 ms time-window while the memory-cue was on screen (**b**; indicated as gray box in **a**) and for the midfrontal P170 peak (**d**; indicated as gray line at 170 ms in **c**). Topographic T-map displaying the difference between trial-groups during memory-cue presentation (**e**; time-window indicated as gray box in **a**). Topographic T-map displaying the difference between correct- and incorrect trials during memory-cue presentation (**f**) and immediately following cue presentation (**g**), asterisks indicate significant differences above electrodes (*p* < .05 cluster-corrected).

As can be seen in Fig. 5a and b, a memory-cue-induced CA component is not present during early learning (first 4 trials), but emerges during the second 4 trials (pairwise comparison Trial-group: T(18) = 2.2; *p* = 0.04). No significant learning-associated effect was observed for the P170-peak (T(18) = 1.16; *p* = 0.26 ns.; see Fig. 5c and d). However, note that the current density T-map displaying the difference between trial-groups during memory-cue presentation (averaged across the time-window as indicated by the gray box in Fig. 5a), shows a negative modulation of midfrontal activity centered around the Afz-Fz electrodes (as well as a positive modulation across right temporal channels; see Fig. 5e).

Next, in a more explorative investigation, we directly contrasted correct- versus incorrect trials (across trial-groups) both during cue-presentation, as well as immediately following cue-offset. Post-cue analysis was performed by averaging responses across a 500 ms time-window following cue-offset, when visualization of the memorized colour-array is expected to occur, in order to respond to the task (average response time is 1500 ms, see Supplementary Figure S4). While during cue presentation some generally enhanced activity is observed particularly around (right) frontal channels for correct- compared to incorrect trials (see Fig. 5f), significant greater activity is observed following cue-offset around posterior central-parietal- and right temporal channels (see Fig. 5g; *p* < 0.05 cluster-corrected). Potentially, the parietal activity facilitates explicit visualization of the memorized array, required in order to correctly make the colour-match judgement (akin to explicit recollection in recognition memory as reported in previous studies^16–25,30,38^. This may occur through interactions with MTL- and occipital regions^30^ (see also “Discussion” section).

### EEG analysis: Longer-term visual memory consolidation reflected in midfrontal P170 and FN400 modulation during the recognition task

In summary and in line with the predictions based on previous studies^1,2^, we observed a WM-specific modulation of the midfrontal P170 component, which became more pronounced with learning. Furthermore, we show that a sustained modulation of the midfrontal component during the WM delay correlates with task performance during the WM task itself *and* with task performance during the subsequent post-hoc recognition task. These findings support the proposition that the midfrontal activity modulation during WM maintenance, reflects recruitment of an LTM-facilitating system.

To further investigate this, we tested whether visual memory consolidation was manifested as midfrontal response modulation upon re-exposure to the colour-arrays^10^, by recording the EEG signal while participants were performing the post-hoc recognition task. Modulatory influences on the neural response were assessed with special focus on the (prolonged) P170 peak (see “Materials and methods” section) and also on a later (FN400) component linked to familiarity recognition ^13^ (and for a review see^16,31^).

**Figure 6:**
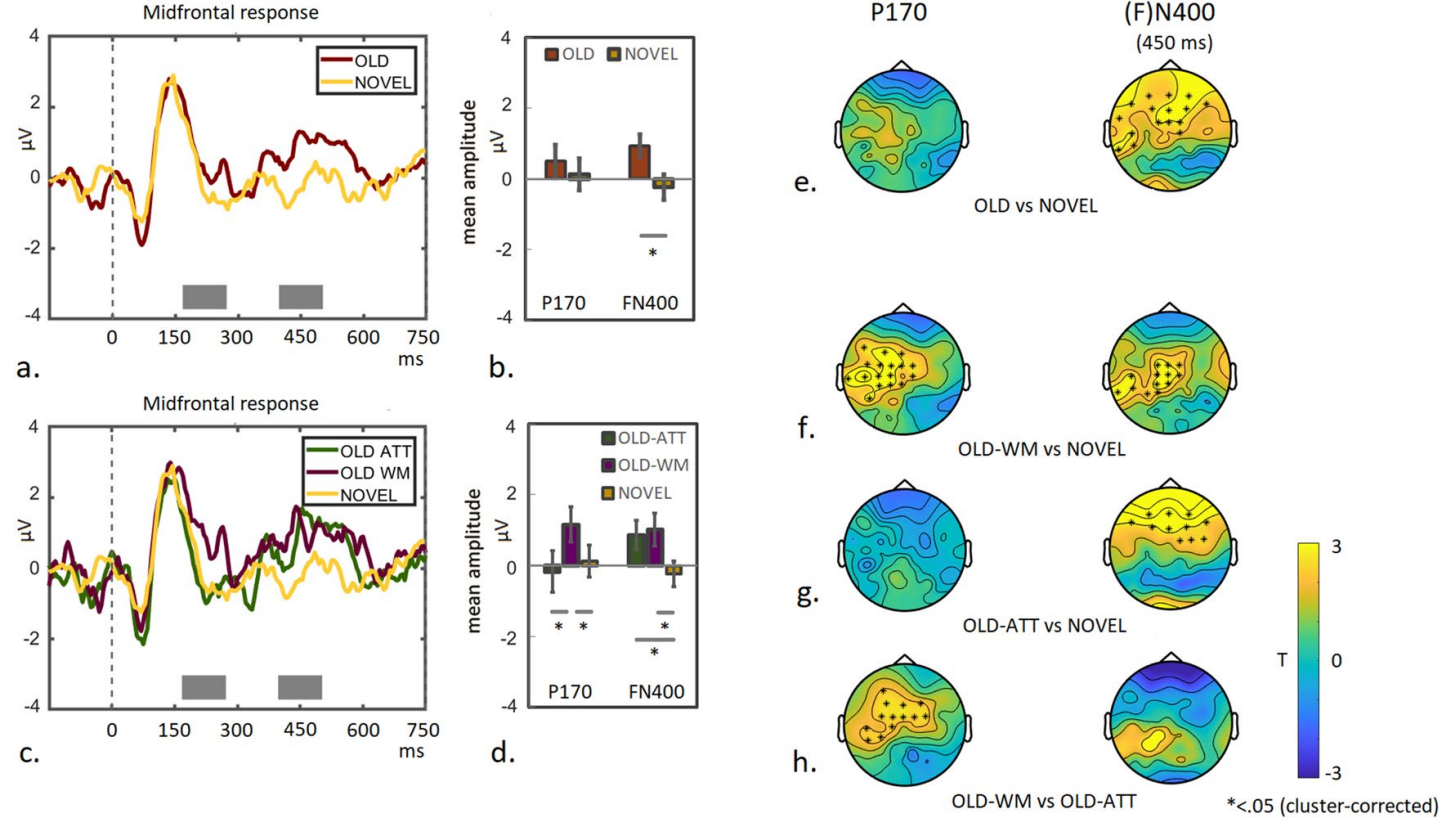
P170 and FN400 modulation and associated topographic activity distributions during the post-hoc recognition task. (**a**) The midfrontal response is shown for 750 ms post array-onset during the recognition task, for previously shown (OLD) and newly created colour-arrays (NOVEL). Mean amplitudes of the P170 (main peak and late modulation) and FN400 component (indicated by gray boxes in **a**) are plotted per condition (**b**). (**c**) The midfrontal response is shown for 750 ms post array-onset for all conditions separately, ie. colour-arrays previously shown during the ATT- or WM condition (OLD-ATT and OLD-WM), or newly created colour-arrays (NOVEL). Mean amplitudes of the P170- and FN400 components (indicated by gray boxes in **c**) are plotted per condition (**d**). (**e**) Topographic T-maps of between-condition differences are shown for the OLD- versus NOVEL contrast for the time-window corresponding to the P170 component (left panel), and the late FN400 peak (450 ms; right panel). Strong positive modulation is visible around midfrontal channels at 450 ms, which corresponds to the described pattern of the FN400 component, but also extends posteriorly to central- and left temporal electrodes. Topographic T-maps displaying between-condition differences are shown per time-window for the OLD-WM vs NOVEL contrast (**f**) the OLD-ATT vs NOVEL contrast (**g**) and the OLD-WM vs OLD-ATT contrast (**h**). Maps for the P170 time-window are shown in left panels and maps for the FN400 peak (450 ms) in right panels. Asterisks indicate significant differences (*p* < .05 cluster-corrected). These results indicate that re-exposure to colour-arrays which previously had been shown during WM-trials (deep learning) but not to those shown during ATT-trials (shallow learning), leads to early activation (< 300 ms) of a recognition-memory network. This is manifested as positive modulation of midfrontal activity (P170), together with posterior central and left-temporal regions. Both OLD categories induce a late FN400 response compared to NOVEL, however with distinctly different associated signal distribution patterns between the two OLD conditions.

Figure 6a shows the midfrontal response to colour-arrays (n = 19), that had either previously been shown during the main task (OLD) or were newly created (NOVEL). The P170- and the late midfrontal FN400 component were averaged for each condition (time-windows are indicated by gray boxes in Fig. 6a) and plotted (see Fig. 6b). A 2 × 2 ANOVA with Repetition (OLD vs NOVEL) and Component as factors, confirmed a Repetition × Component interaction (F(1,18) = 5.24 *p* = 0.034). Pairwise comparison between the repetition-conditions for each component yielded no significant difference for the main P170 signal (T(18) = 1.16; *p* = 0.26), but did so for the FN400 component (T(18) = 2.53 *p* = 0.021). Scalp distributions of the between-condition differences during P170 time-window and the peak of the FN400 signal (450 ms) are shown in Fig. 6e. No clear modulation can be observed during the P170 time-window, when comparing OLD- to NOVEL trials (left panel), but a clear positive signal modulation is visible around midfrontal channels at 450 ms (right panel). The activity distribution corresponds to the described pattern of the FN400 component (see^16^ for a review), but also extends posteriorly to central- and left temporal electrodes (asterisks indicate *p* < 0.05; cluster-corrected).

Note that the OLD-condition includes colour-arrays that were previously shown either during working memory- (WM) or attention- (ATT) trials. Based on the idea that an LTM-supporting system was recruited to sustain memory during the delay-phase of the WM task, which would not have taken place during the ATT trials, we expected a differential neural signature between these categories upon re-exposure.

The data are therefore plotted again with the repeated arrays split into two trial conditions, depending whether they had previously been shown during the WM- or the ATT trials or were newly created (NOVEL). Inspecting the EEG-traces, a prolonged modulation of the P170 component can be observed in response to OLD arrays previously presented during the WM-condition in the main experiment (OLD-WM), but not to OLD arrays presented during the ATT-condition in the main experiment (OLD-ATT), or the NOVEL condition. In contrast, the later FN400 component seems enhanced for both repeated (OLD) conditions, compared to the NOVEL condition. Mean amplitudes of the P170- and the FN400 components (indicated by gray boxes in a) are plotted per condition in Fig. 6d. A 3 × 2 ANOVA with Repetition (OLD-ATT, OLD-WM and NOVEL) and Component (P170 and FN400) as factors, indeed confirmed a significant Repetition x Component interaction (F(2,36) = 4.09 *p* = 0.025). Direct comparison confirmed a significant difference in the P170 component between the OLD-WM- and the NOVEL condition (T(18) = 2.96; *p* = 0.008), but not between the OLD-ATT and the NOVEL condition (T(18) =  − 0.43 *p* = 0.68 ns). Direct comparison between the OLD-WM- and the OLD-ATT conditions, also revealed a significant difference (T18) = 2.53 *p* = 0.021). This finding is in line with our prediction that specifically re-exposure to colour-arrays, which had previously been held in WM, would reveal longer-term neural plasticity in the midfrontal P170 component. In contrast, no difference in neural response was observed between the OLD-WM and the OLD-ATT conditions for the FN400 component (T(18) =  − 0.24 *p* = 0.81), but both conditions induced enhanced responses compared to the NOVEL condition (OLD-WM vs NOVEL T(18) = 2.17 *p* = 0.043; OLD-ATT vs NOVEL T(18) = 2.01 *p* = 0.06). Similar effects were observed when repeating this analysis for the participants with recorded behavioural responses only (n = 13; see Supplementary Figure S8). This analysis also confirmed an accuracy effect for the FN400 component^16^.

A distinct difference in topographic pattern becomes apparent between the two OLD-conditions (OLD-WM and OLD-ATT) when comparing these with the NOVEL-condition. Contrasting OLD-WM to NOVEL shows profound modulation of signal around midfrontal channels extending to posterior central- and left-temporal channels, during the time-window corresponding to the P170 component (Fig. 6f, left panel). In contrast, no clear differential activity is observed when contrasting OLD-ATT to NOVEL (Fig. 6g, left panel).This pattern is confirmed when contrasting OLD-WM to OLD-ATT directly (see Fig. 6h, left panel; asterisks *p* < 0.05, cluster-corrected).

Also for the peak of the time-window corresponding to the FN400 signal, a striking difference between conditions is visible. The observed positive modulation of the FN400 component for both OLD-conditions (Fig. 6c) seems to arise from different signal distribution patterns, which becomes apparent when contrasting each OLD condition to NOVEL: in case of the OLD-WM condition, the enhanced signal around midfrontal channels forms the frontal edge of a more posteriorly centered activity pattern around central-parietal and left temporal channels (see Fig. 6f, right panel). In contrast, when comparing the OLD-ATT condition to NOVEL, the enhanced midfrontal signal is part of a more frontal distribution (see Fig. 6g, right panel; note that some activation around occipital regions is observed as well). A direct comparison confirms this difference (see Fig. 6h, right panel; see also Supplementary Figure S9).

In summary, a pattern emerges which suggests that specifically re-exposure to colour-arrays previously shown during the WM task, leads to early (< 300 ms) activation of a recognition-memory network. This is manifested as positive modulation of the midfrontal P170 signal, extending to posterior central- and left-temporal regions. The late modulation of the midfrontal FN400 component, associated with conscious recognition (familiarity; see ^13,15,16,31^), is present for both categories of repeated colour-arrays, irrespective whether they had previously undergone deep learning (WM) or shallow learning (ATT), but differences were observed in the associated activity-distribution patterns (see also “Discussion” section and Supplementary Figure S9).

## Discussion

The current study investigated whether the gradual strengthening of a multi-item abstract visual memory, which is explicitly probed, concurs with a shift of parietal-to-midfrontal neural activity, as was previously shown for the memory of a single visual-search target (probed by the *implicit* memory measure of response speed)^1,2^. In addition, we also investigated the hypothesis that this shift in neural correlates could signify the recruitment of a longer-term memory supporting neural network.

Our results reveal a learning-associated decline in parietal CDA during repeated memorizing of a six-item colour-array. However, even during the early learning phase, the CDA is, on average, only briefly observed and not throughout the whole WM-delay period. This could indicate that maintenance of the memory trace is just briefly and not sufficiently sustained by parietal activity even during these early learning trials. Alternatively, it is possible that the memory representation becomes less lateralized throughout the delay and therefore hard to detect by measuring the CDA. Interestingly, contrasting correct- to incorrect trials suggests that the CDA component is beneficial (to some extend) during early learning, but detrimental during late learning.

Our experimental paradigm was deliberately designed to promote an interaction between a fronto-parietal WM-network and MTL-hippocampal regions. We expected that the combination of a beyond-WM-capacity set-size and the spatial context of our task, would enhance reliance on MTL-hippocampal structures (see^8^). Due to the large set size, we expected that the memory trace could not be sustained by parietal visual WM mechanisms alone and that an extended memory network would need to be recruited. It seems indeed the case that the parietal CDA response did not sustain the visual memory sufficiently and a WM-specific modulation of the midfrontal P170 component (compared to the attention condition), became more prominent as learning progressed. This modulation is similar to what was previously described in the repeated search paradigm^1,2^, which was interpreted as a transfer of the memorandum (search target) to an LTM storage system. This interpretation was motivated by previous studies which demonstrated modulation of this component during long-term perceptual memory priming processes^10^. In our task no significant correlation between modulation of the P170 component and task-accuracy is observed. However, we find that the level of negative modulation of the midfrontal response during the delay period is predictive for WM accuracy when learning progresses (and a transition from WM to short-term memory occurs). This suggests that *sustained modulation* of midfrontal activity is involved in maintaining the memory trace during the WM delay.

Interestingly, whereas the CDA component gradually decreases during the WM-delay, a learning-associated CA component slowly emerges during *recollection/visualization* of the memorized colour-array, prompted by the combined colour-spatial cue following the WM-delay. In fact, successful recollection is associated with significant activation around posterior central-parietal- and right temporal channels following memory-cue offset, which we propose might reflect explicit visualization needed to respond to the task. Albeit different from recognition memory, this observation could potentially be compared to previous reports of a late parietal component during recollection in LTM^13,16^, which might be more right-lateralised for pictures as compared to words^39^. As Rugg et al. demonstrated and corroborated by multiple studies since, a late parietal component is specifically observed during *explicit, vivid recollection* of a memorandum^16–25,38^.

In addition to this, our results provide support for an involvement of contralateral parietal activity in *encoding* of the colour-array, as a positive EEG-behaviour correlation with performance on the post-hoc recognition task is observed. Qualitatively, the C(D)A narrows in duration when learning progresses and is still (and only) present during array-presentation. This may indicate involvement in a (gradually) more efficient encoding process.

While many studies indicate a role for fronto-parietal regions in memory-encoding^40,41^, our findings suggest that parietal CA reflects a specific mechanism, which can be dissociated from selective attention (as no CA activity was observed during the attention condition). This mechanism may involve processes that promote LTM by strengthening inter-item associations^42,43^, and/or spatial binding, possibly through interaction with MTL- and hippocampal structures^30,44–47^.

It could be speculated that the observed parietal CA in our study, may serve to bring visual information in a privileged (stable) mental state or buffer^28,48–52^, initially to facilitate encoding (and maintenance) of visual input and subsequently to visualize the detailed, spatial context of the memorized information during recollection. The sustained parietal activity during these processes may facilitate information transfer to (and from) MTL/Occipital regions^5,28,30,49–55^, as suggested by the observed modulation of activity around (right) temporal channels during WM maintenance and particularly also during successful recollection/visualization.

Importantly, the averaged midfrontal amplitude during WM-delay correlated with performance accuracy in the subsequent post-hoc recognition task. This suggests that the sustained modulation of the midfrontal component during WM indeed reflects recruitment of an LTM-supporting network^1,2^ which contributes to WM-maintenance during the delay phase.

Surprisingly, a positive EEG-behaviour correlation was observed during early learning trials, both for task accuracy in the WM- and the post-hoc recognition task. Perhaps midfrontal activity subserves different underlying mechanisms and memory is first actively consolidated during early learning trials, while merely protected from interference during late learning trials.

Visual memory consolidation was not only shown on the behavioural level, but also lasting influences on the midfrontal response were demonstrated upon re-exposure to previously presented colour-arrays during the recognition task. Importantly, modulation in the P170 component was observed during trials in which colour-arrays were correctly recognized as ‘old’, but only for arrays that had previously been shown during WM- as opposed to ATT-trials. This confirms our expectation that specifically re-exposure to colour-arrays which had previously been held in WM, sustained by an LTM-supporting network, would reveal long-lasting modulation within this network. These findings further support the proposition that P170 modulation during the WM task is indicative of the recruitment of an LTM supporting network during WM maintenance.

Interestingly, a later midfrontal component attributed to familiarity recognition memory, the FN400, shows a modulation for both OLD categories, irrespective whether arrays had previously been shown during WM- or ATT-trials. However, associated topographic activity distribution maps suggest that different underlying mechanisms may have given rise to the FN400 familiarity signal. While arrays previously held in WM (deep learning) induced an activity pattern extending to posterior centro-parietal and (left) temporal regions, a more frontal activity distribution was observed for responses to arrays that were previously merely attended to (shallow learning). The pattern induced by the WM category may therefore be more a combination of the FN400 and the N400 signal as described in previous studies^13–16^ and reflect visual concept-formation.

In general, a pattern emerges from our results, which suggests that specifically re-exposure to colour-arrays which had previously been shown during the WM task, leads to early activation (< 300 ms) of a recognition-memory network, manifested as positive modulation of midfrontal activity (P170) and recruitment of posterior left-temporal and occipito-parietal regions. Furthermore, a modulation across conditions of the late midfrontal FN400 component, which is generally associated with conscious recognition (familiarity), seems part of a wider network and potentially reflects a connection between a recognition-memory network and other more frontal regions.

The source of the midfrontal activity cannot be properly assessed with EEG recordings, but is likely to be located in the ventromedial prefrontal cortex, which has been implicated in memory encoding by multiple studies^56–58^ (and see^59^ for a review). Further analysis of the nature of the signal could clarify a potential link with midfrontal theta oscillations, which have been associated with maintenance during working memory^26^ and information-transfer to MTL/hippocampal regions during encoding- and retrieval processes in long-term memory (see fi.^27^ for a review). Modulation of the P170 peak itself might reflect a memory-specific phase-reset mechanism of midfrontal theta-oscillations, which prepares the system for memory encoding^26^.

## Conclusion

In conclusion, the present study provides evidence for a gradually stronger recruitment of an LTM-supporting neural network, when a multi-item visual image is repeatedly held in WM. This is indexed by (sustained) modulation of midfrontal activity during encoding and WM maintenance. In addition, enhanced parietal CA and activation of right temporal regions is observed during gradually more successful recollection/visualization of memorized items following the WM delay. In contrast, a declining role for parietal CDA during WM maintenance is visible over time. Finally, recruitment of an LTM-supporting network during WM-maintenance is reflected in longer-lasting plasticity within this memory-network, which becomes apparent as late modulation of the midfrontal P170 component, together with enhanced left-temporal activity as well as later central-parietal activation, upon re-exposure to the memorized images.

## Supplementary Information

Supplementary Information.

## Data Availability

The datasets generated- and/or analysed during the current study are not publicly available due to ongoing analysis for a follow-up study, but are available from the corresponding author upon reasonable request.
