# Supplementary material for: Recruitment of a long-term memory supporting neural network during repeated maintenance of a multi-item abstract visual image in working memory
Source: Sci Rep. 2022 Jan 12;12:575. doi: 10.1038/s41598-021-04384-4 (PMC8755800; doi:10.1038/s41598-021-04384-4)
Supplement: Supplementary file 1 — Supplementary Information. [file 41598_2021_4384_MOESM1_ESM.pdf]

Supplementary information:

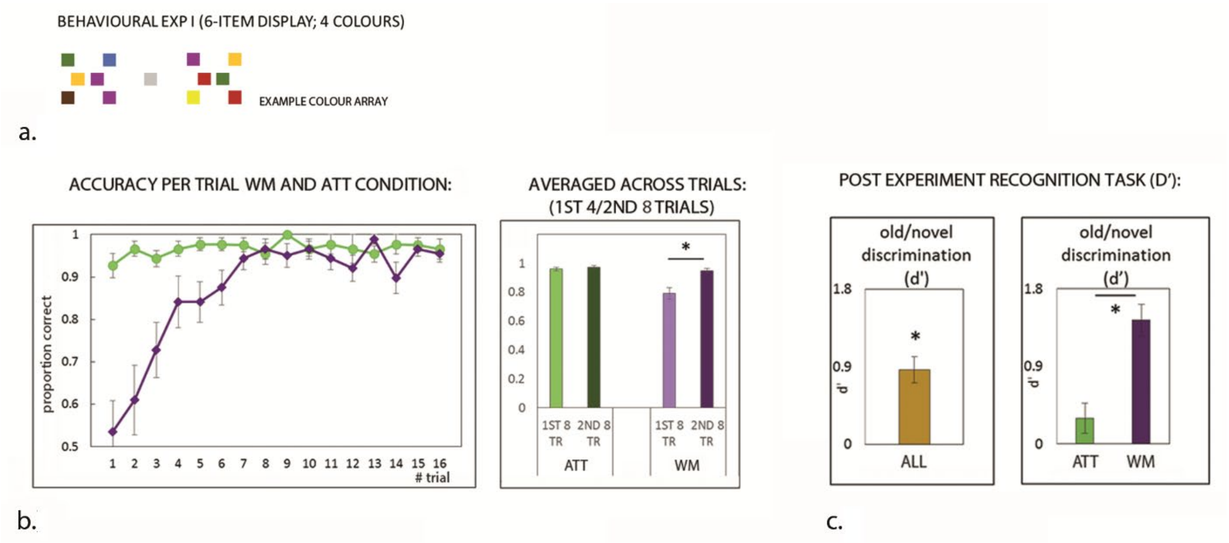

Fig. S1 behavioural experiment 1.

a.)

Example of a colour-array used in pilot experiment 1. Four different colours were randomly drawn (separately for each hemifield) from a pool of 8 highly discriminable colours and allocated to each of 6 squares of an array, with two colours being repeated.

As in the paradigm used for the EEG experiment, the two colour-arrays were bilaterally presented with one hemifield cued for covert attention at the beginning of a testing block. During this behavioural experiment, the same array-combination was repeated for 16 trials in one testing block (this was reduced to 8 during the EEG paradigm). As during the EEG paradigm, spatial locations of all squares would remain visible as white placeholders during stimulus intervals throughout the testing block. Participants had to match a central colour probe with the colour at one highlighted square in the array, either while the colour-array was still present on screen (ATT condition) or following a 2.5 second delay (WM condition). As in the EEG paradigm, the main experiment was followed by a post-hoc recognition task.

During this task, all colour-arrays that had been presented during the main task on the attended hemifield were shown again, randomly intermixed with novel array configurations made up out of the same colour pool. Participants had to indicate whether they recognized the presented arrays with a confidence above 60%.

b.)

Mean proportion correct scores are plotted for 16 consecutive trials for the ATT and the WM condition (n=11). A gradual increase in accuracy during the WM condition reached ceiling around the 8th trial, while performance for the attention condition was around ceiling throughout the testing block. In the right panel, the accuracy scores for both conditions are plotted, averaged across the first- and the second eight trials. A learning effect was observed for the WM condition between trial groups which was not observed in the ATT condition (Interaction Task x Trial Group $F(1,10)=23,7$ $p<.001$; Effect Trial Group ATT ($T(10)=-1.2$ $p=.26$ ns.; Effect Trial Group WM ($T(10)=-5.7$ $p<.001$).

c.)

Accuracy scores on the post-hoc recognition task are plotted as d' scores across conditions (left panel) and separated for colour-arrays, that had previously been shown during the ATT or the WM condition (right panel). The d' score across conditions was greater than zero ($T(10)=5.7$ $p<.001$), which indicates a long-term consolidation of the memory-trace. Note that the colour-arrays that had been presented during the WM condition in the main experiment were significantly better remembered than those presented during the ATT condition ($T(10)=-6.29$ $p<.001$).

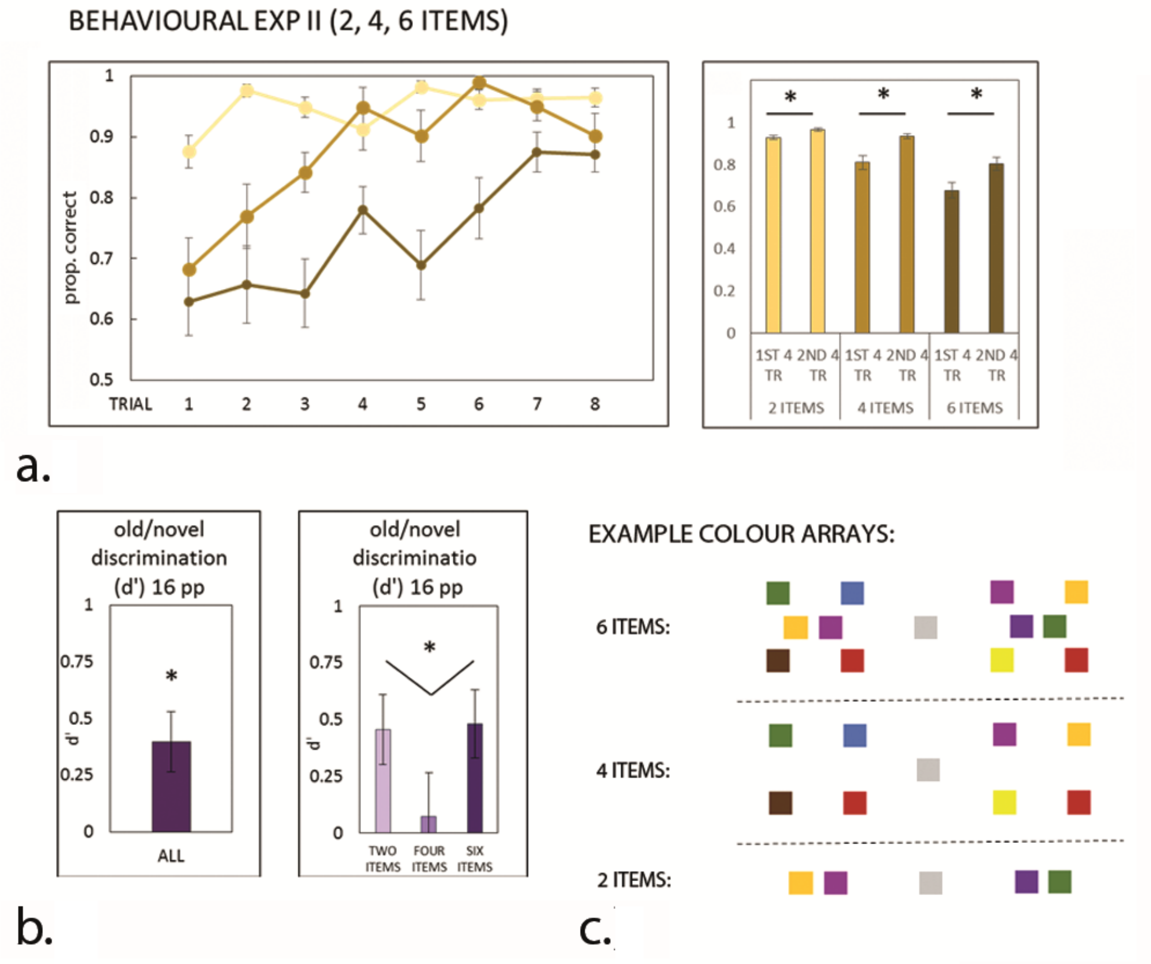

Fig. S2 behavioural experiment 2.

In the second pilot experiment, a similar visual WM paradigm was used as in behavioural experiment 1, except that three set-size conditions were tested. Example of colour-arrays used in behavioural experiment 2. Six, four or two different colours were randomly drawn (separately for each hemifield) from a pool of 8 highly discriminable colours and allocated to each square of a six, four or two square-array (shown in c), depending on the WM condition of the testing block. These set-sizes were chosen deliberately around the visual WM capacity limit (around 4) as it was hypothesized that a set-size beyond the capacity limit (6) would enforce a strategy that relied more on LTM-supporting brain-structures. Importantly, we expect that an enhanced reliance on the LTM system for holding a mental representation online during the

WM condition, would result in a stronger memory consolidation. This should be reflected in a higher accuracy score during a post-test recognition task.

We found indeed support for this hypothesis with 2- and 6-item arrays being better remembered in a post-hoc recognition task than 4-item arrays (shown in b; quadratic effect d' between ITEM nr. conditions $F(1,15)=8,1$ $p=.012$; Main effect d' across all ITEM nr. conditions $T(15)=2.7$ $p=.016$).

The different learning curves (n=16) are shown for the 2,4 and 6-item arrays during the 8-trial visual WM task in a, left panel. Averaging across first- and second four trials showed significant learning effects for all conditions (a, right panel; $p<.005$).

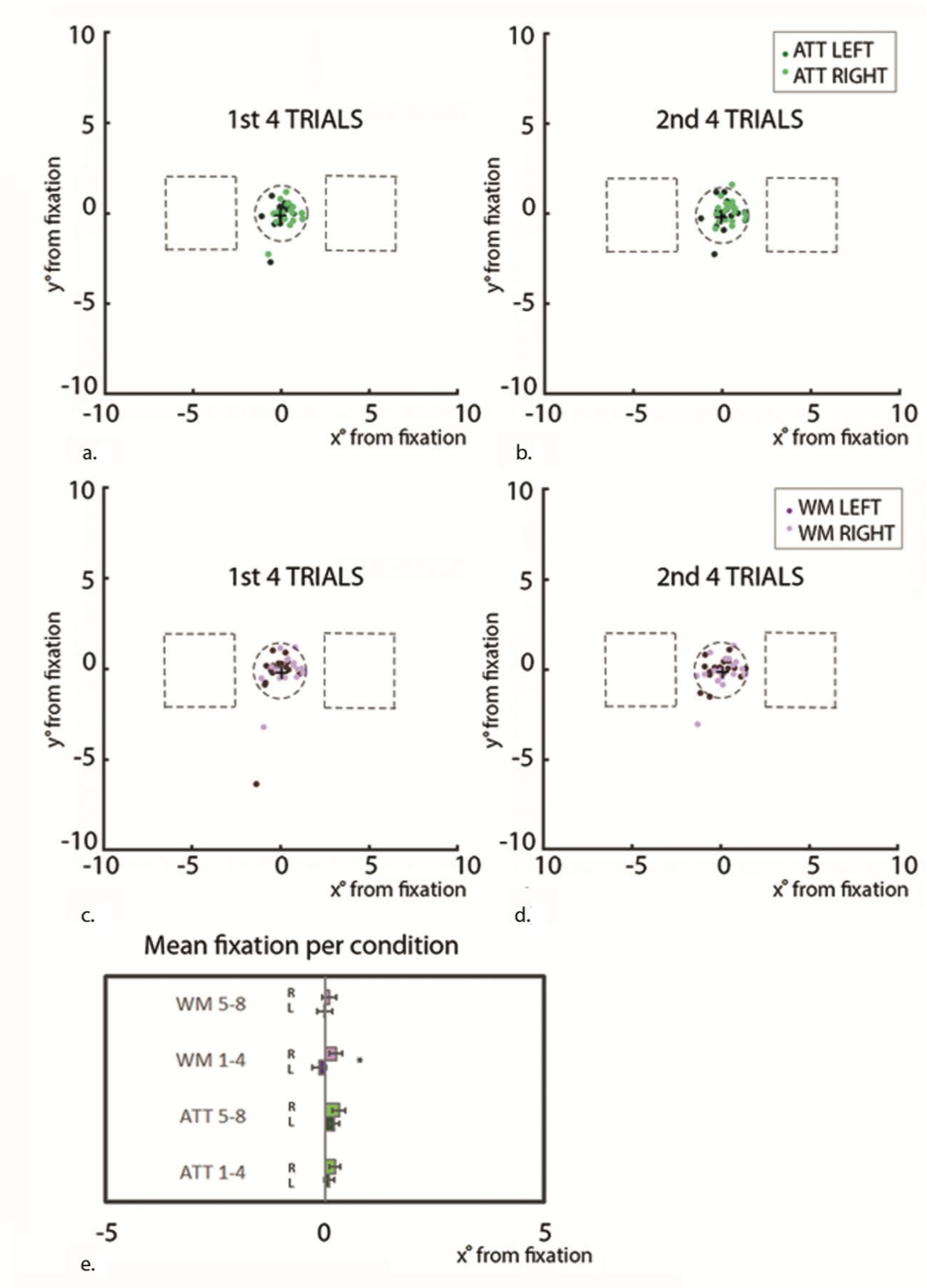

Fig. S3 Eye fixation.

Mean fixation coordinates were within 1.5º from the center for all participants (indicated by the dashed circle) in all conditions (a-d). Dashed squares indicate the location of the colour-arrays

on screen. Across participants a slight hemifield bias was observed during the first 4 trials of the WM condition, and not in any other condition (e; R=right hemifield trials; L=left hemifield trials). The small magnitude of this (<.3° on average) is not thought to have any substantial impact on the CDA measurements (see methods).

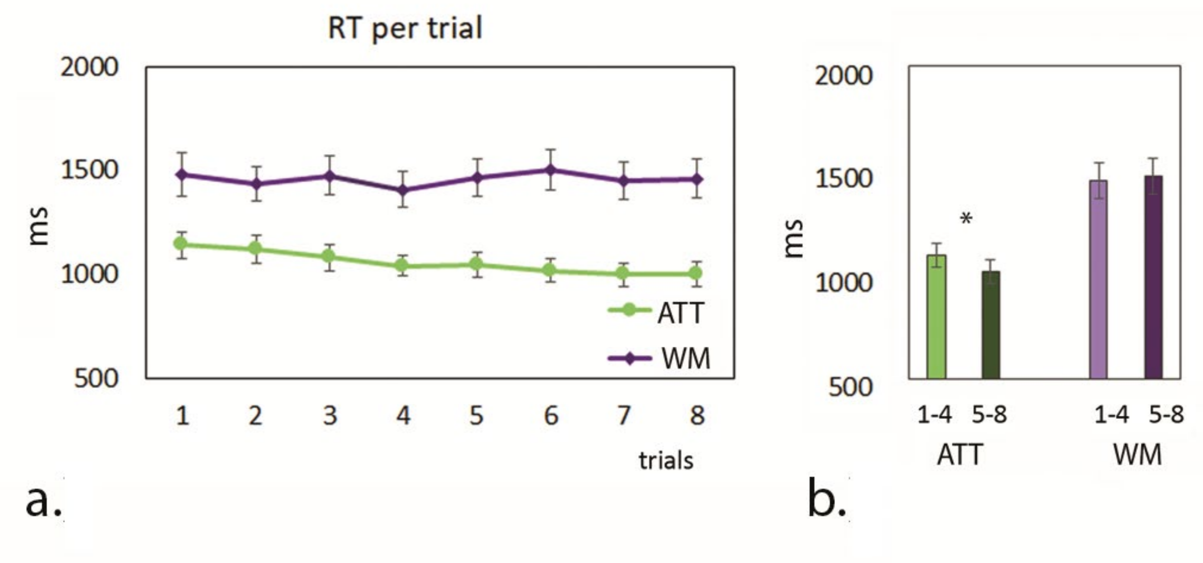

Fig. S4 Reaction Times.

Mean RT scores (calculated from array-onset (ATT condition) or cue-onset (WM condition) are plotted for 8 consecutive trials each condition. RT's got gradually faster for the ATT condition but did not change across trials for the WM condition. Overall RTs were faster for the ATT condition b.) Plotted are the RT scores for both conditions, averaged across the first- and the second four trials (Main effect Task $F(1,19)=42,1$ $p<.001$; Main effect Trial Group $F(1,19)=4.71$ $p=.043$; Interaction Task x Trial Group $F(1,19)=9$ $p=.007$) (ATT Trial Group $T(20)=5.5$ $p<.001$; WM Trial Group $T(20)=-.79$ $p=.44$).

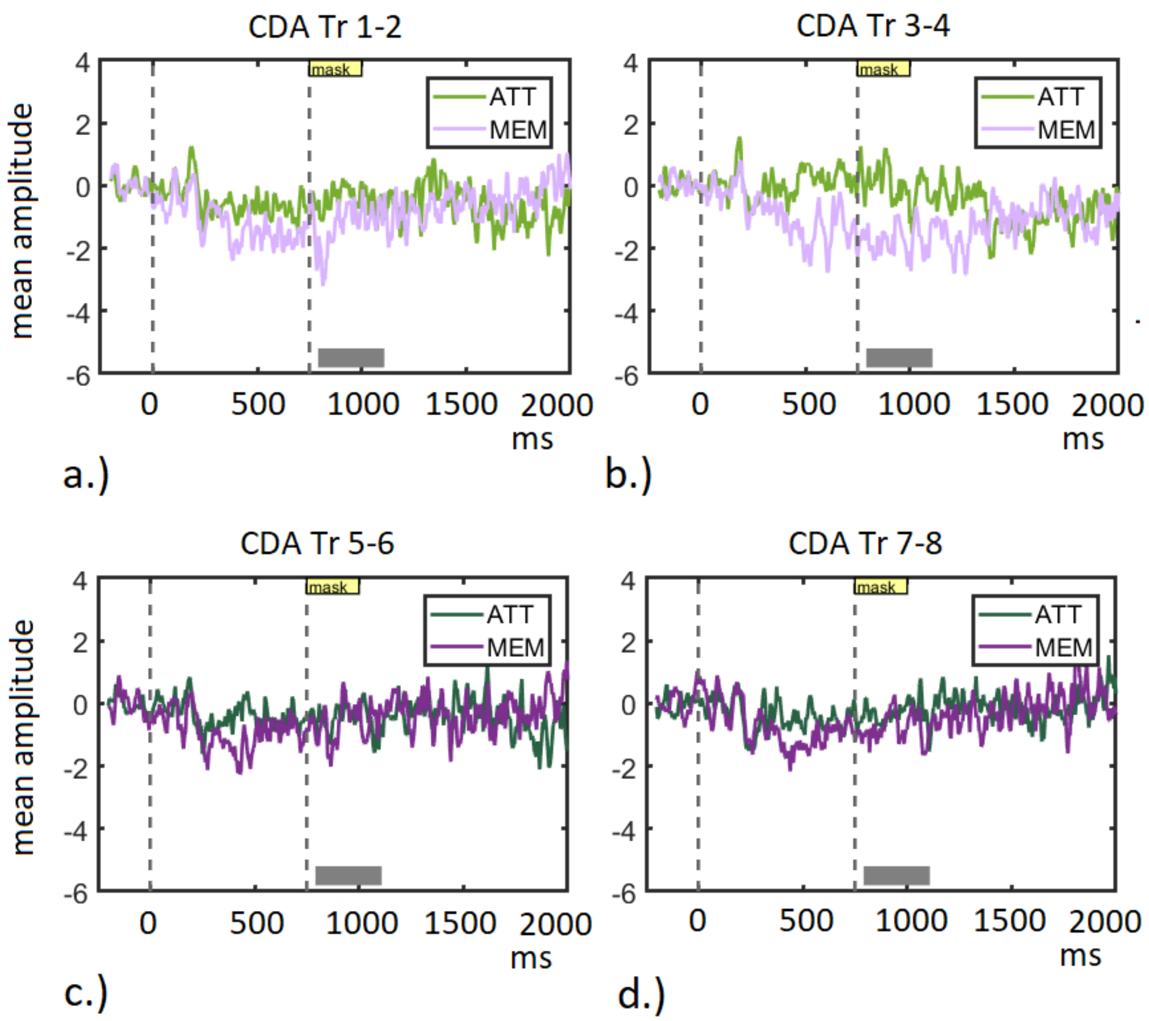

Fig. S5 Parietal contralateral activity during encoding and WM maintenance split into 4 groups of averages across 2 trials.

C(D)A components are plotted as difference waves between contra- and ipsilateral activity per task-condition (ATT/WM), from the moment of colour-array onset and throughout 2000ms of the WM delay period averaged across activity during the first two trials (TR 1-2) (a), the second two trials (TR 3-4) (b), the third two trials (TR 5-6) (c) and the fourth two trials (TR 7-8) (d). An emerging CDA during the first two trials (TR 1-2) grows most prominent and extending till ca. half a second into the delay during the consecutive second two trials (TR 3-4) and shrinks to a short-lasting signal during cue-presentation during the last two trial-groups (resp TR 5-6 and TR 7-8).

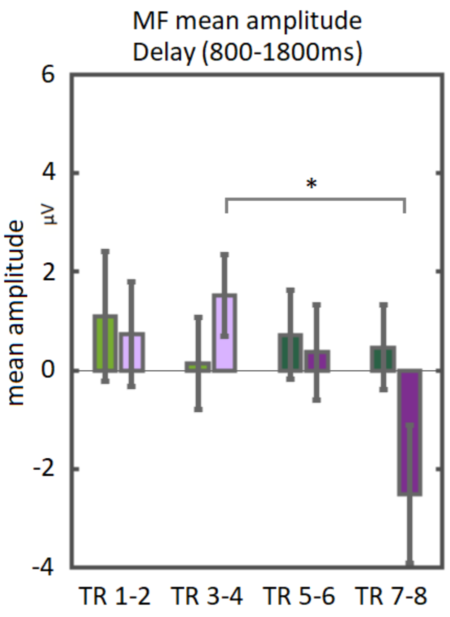

Fig. S6 Midfrontal responses per 2 consecutive trials during WM delay, averaged across 1000 ms post array-offset.

Plotted are the mean amplitudes of the midfrontal responses averaged across a 1000 ms time-window immediately following array-offset per task-condition and per trial-group of two trials. A 2x4 ANOVA (Task, Trial-group) revealed no significant interactions between groups ($F(3,72)=1.65$; $p=.19$) and also no significant post-hoc pairwise interactions ($p>.15$ Tukey-Kramer correction for multiple comparison). As explained in the main text however, direct comparison between tasks is slightly problematic as the ATT-task requires a response immediately following display offset. A separate 1x4 ANOVA for the WM condition only was therefore performed as well. This yielded a marginally significant interaction effect between groups ($F(3,72)=2.66$ $p=.055$). Post-hoc pairwise comparison revealed significant differences for the WM-condition between trial-bin 1(TR1-2) and trial-bin 4(TR 7-8) ($p=.037$) and trial-bin 2 (TR3-4) and trial-bin4 ($p=.01$).

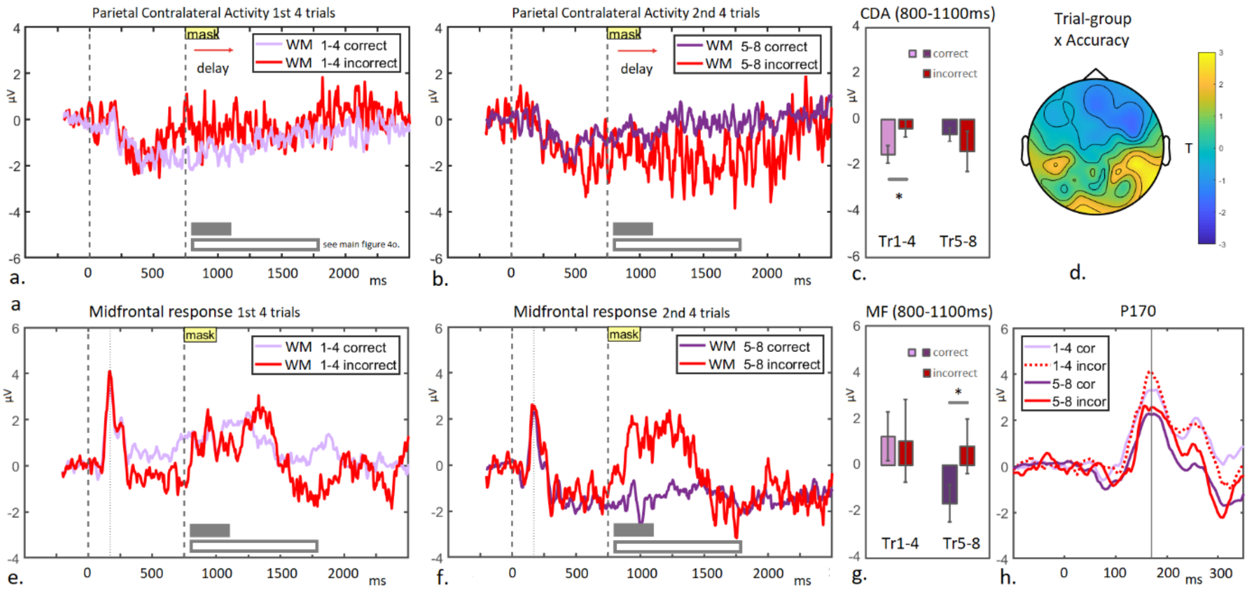

Fig.S7 Parietal contralateral activity and midfrontal response during encoding and WM maintenance for correct- and incorrect WM-trials.

C(D)A components are plotted as difference waves between contra- and ipsilateral activity for correct- and incorrect trials of the WM-condition, averaged across activity during the first four trials (TR 1-4) (a) and the second four trials (TR 5-8) (b).

Mean amplitude averaged across 300ms post display-offset (c; see grey bar in figure).

Interestingly, the incorrect trials show an opposite pattern from the correct-trials. While CDA is observed correct- but not for incorrect trials during TR1-4, during TR5-8 it was observed for incorrect- but no longer for correct trials (($F(1,18)=4.2$ $p=.056$; pairwise comparison correct vs incorrect trials TR1-4 $T(18)=-2.93$; $p=.009$; TR5-8 $T(18)=.8$ $p=.45$ns. but note that this contrast is significant when averaging across a longer 1sec time period (see main text)).

Current-density map displays interaction between Trial-group and Accuracy for this 300ms time-window confirms a pattern of negative modulation of activity recorded at the midfrontal electrodes and also showed an enhanced activity above right central-parietal and temporal electrodes (d.).

The midfrontal responses for correct- and incorrect trials of the WM-condition are plotted from colour-array onset and throughout the delay period averaged across activity during the first four

trials (TR 1-4) (e) and the the second four trials (TR 5-8) (f). Mean amplitude averaged across 300ms post display-offset (g; see grey bar in figure). The response pattern for incorrect trials seems quite similar during TR1-4 and TR5-8, with a steep rise immediately following array-offset. An ANOVA shows no significant interaction ($F(1,18)=1.84$; $p=.192$), but pairwise comparison between correct- and incorrect trials is significant during TR5-8 ($T(18)=-2.2$ $p=.04$ and not during TR1-4 ($T(18)=.23$; $p=.82$).

The midfrontal P170 responses are plotted separated for conditions for the midfrontal during 350 ms following colour-array onset (h.). No significant effects were found (see main text).

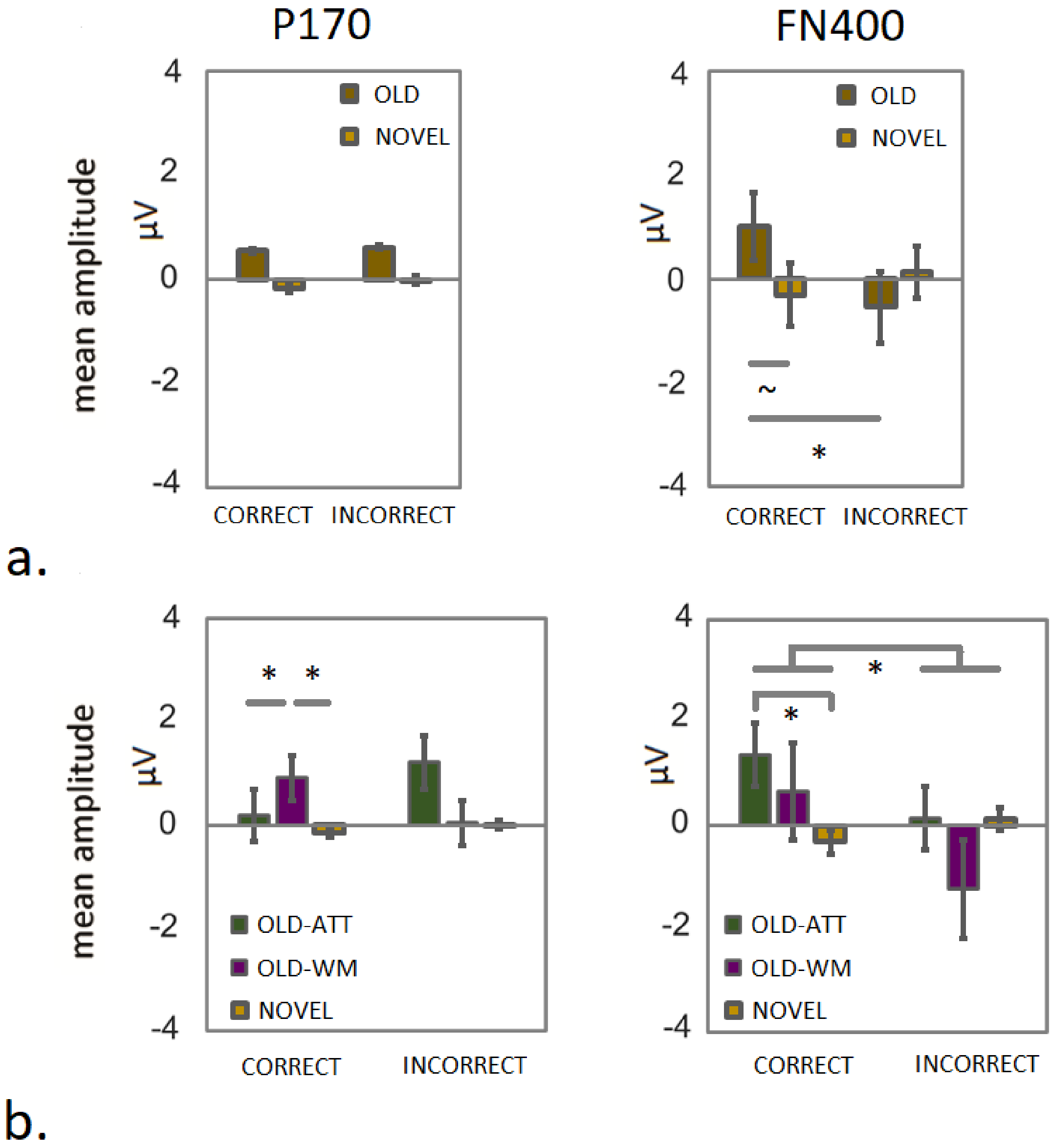

Fig.S8 The P170 and FN400 mean amplitudes in response to old/novel colour-arrays during the recognition task separated for repetition-condition and accuracy (n=13).

a.) Plotted are the mean amplitudes of the P170 and FN400 components during the post-hoc recognition task in response to old- and novel colour-arrays (Repetiton), separated by accuracy (n=13). A 2x2x2 ANOVA indicated a marginal Component x Repetition x Accuracy interaction effect ($F(2,12)=3.71$ $p=.078$).

A 2x2 ANOVA for the P170 response separately, showed no Repetition x Accuracy interaction ($F(1,12)=.015$ $p=.91$) but a marginal main effect of Repetition ($F(1,12)=3.22$ $p=.098$). Pairwise comparison indicated non-significant differences between OLD-CORRECT and NOVEL-CORRECT ($T(1,12)= 1.15$ $p= .16$) and OLD-INCORRECT and NOVEL-INCORRECT ($T(1,12)= 1.14$ $p= .28$).

A 2x2 ANOVA for the FN400 component separately, revealed a marginal Repetition x Accuracy interaction ($F(1,12)=3.31$ $p=.094$). This reflected a greater response for OLD-CORRECT vs OLD-INCORRECT ($T(12)= 2.77$ $p=.017$) and no difference between NOVEL-CORRECT vs NOVEL-INCORRECT ($T(12)=-.61$ $p=.56$). In line with the reported analysis including all 19 participants (see Figure 6 in main text), there was a (marginal) difference between OLD-CORRECT and NOVEL-CORRECT ($T(1,12)=2.02$ $p=.066$). There was no significant difference between OLD-INCORRECT and NOVEL-INCORRECT ($T(1,12)=-1.11$ $p=.28$).

b.) The mean amplitudes of the P170 and FN400 components are plotted again for old- and novel colour-arrays, separated by accuracy, but with the old arrays also separated with respect to their category during the main task (OLD-ATT and OLD-WM). A 2x3x2 ANOVA revealed no significant Component x Repetition x Accuracy interaction effect ($F(2,12)= 1.87$ $p= .18$) or main effect of Repetition-condition ($F(2,12)= 1.94$ $p=.17$).

A 2x2 ANOVA for the P170 response separately showed a marginal Repetition x Accuracy interaction ($F(1,12)= 2.87$ $p= .076$) and no main effects ($p>.23$). As for the analysis including all 19 participants (see Figure 6 in main text) pairwise comparison revealed a significant difference between OLD-WM and NOVEL for correct trials ($T(12)= 2.24$ $p=.045$), but not between OLD-ATT and NOVEL ($T(12)= .67$ $p= .52$). Direct comparison between OLD-WM and OLD-ATT also revealed a significant difference ($T(12)= 2.28$ $p= .042$). No significant differences were observed for incorrect trials (OLD-ATT vs NOVEL $T(12)= 1.5$; $p= .16$; OLD-

WM vs NOVEL T(12)= .084 p= .94); OLD-ATT vs OLD-WM T(12)= 1.43 p= .179). Direct comparison between correct- and incorrect trials did not reveal significant effects (OLD-ATT-CORRECT vs OLD-ATT-INCORRECT T(12)= -1.54 p= .15; OLD-WM-CORRECT vs OLD-WM-INCORRECT T(12)= 1.6 p= .14; NOVEL-CORRECT vs NOVEL-INCORRECT T(12)= -.33 p= .745).

A 2x2 ANOVA for the FN400 response separately revealed a significant main effect of accuracy (F(1,12)= 6.45; p= .026), no significant main effect of Repetition-condition (F(1,12)= 2.29 p= .12) or Repetition x Accuracy interaction (F(1,12)= 1.58 p= .23. As for the analysis including all 19 participants (see Figure 6 in main text) pairwise comparison revealed a significant difference between OLD-ATT and NOVEL (T(12)= 2.432 p= .032), however no significant difference was found between OLD-WM and NOVEL (T(12)= 1.22 p= .245). As for the analysis including all 19 participants, no significant difference was observed between OLD-ATT and OLD-WM (T(12)= 1.0 p= .34).

Direct comparison between correct- and incorrect trials revealed a marginal difference between OLD-ATT-CORRECT and OLD-ATT-INCORRECT (T(12)= 2.05 p= .063), no significant difference between OLD-WM-CORRECT and OLD-WM-INCORRECT (T(12)= 1.66 p= .123) and no difference between NOVEL-CORRECT and NOVEL-INCORRECT T(12)= -.61 p= .56).

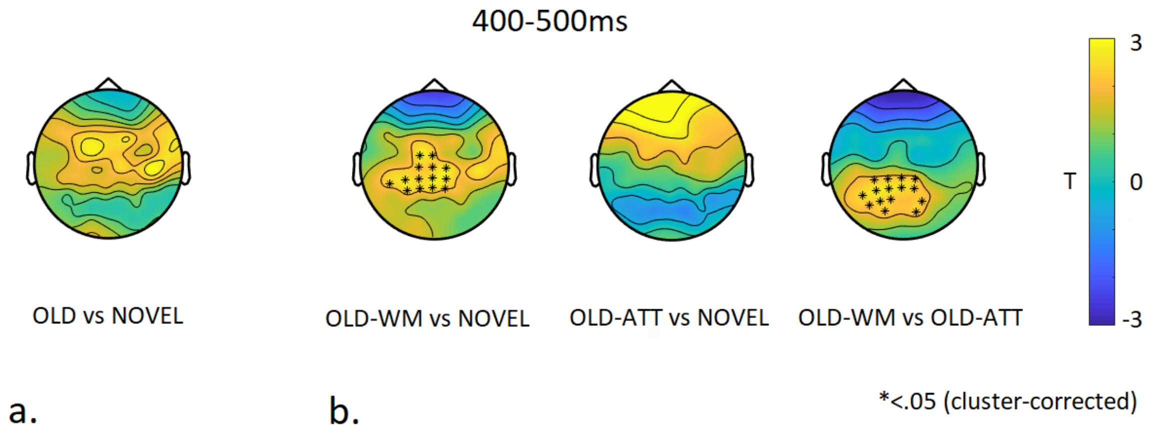

Fig.S9 Topographic T-maps of between-condition differences averaged across the whole time-window corresponding to late FN400 peak (400-500ms).

Topographic T-maps of between-condition differences are shown for OLD vs NOVEL (a) and respectively for OLD-WM vs NOVEL, OLD-ATT vs NOVEL and OLD-WM vs OLD-ATT contrast (b).

A positive modulation is visible around midfrontal channels, which corresponds to the described pattern of the FN400 component, for the OLD vs NOVEL, the OLD-WM vs NOVEL and OLD-ATT vs NOVEL. However, the OLD-WM vs NOVEL contrast shows an activity distribution that extends more posteriorly including central-parietal channels, which has been described for the N400 component[52]. This difference is confirmed when contrasting the OLD-WM and OLD-ATT conditions directly (most right panel). The posterior N400 effect is associated with 'absolute familiarity' (as opposed to relative familiarity) and may indicate that visual concept formation has taken place for the colour-arrays that had previously been held in WM. The FN400 modulation as observed for the OLD-ATT group is likely to rely on a different mechanism and may reflect interactions with other (dorso-) frontal regions. Note that some occipital activity was observed as well during the 450ms peak (see main text), possibly indicating perceptual plasticity in lower visual areas..
